# 3D-printed porous Ti6Al4V scaffolds for long bone repair in animal models: a systematic review

**DOI:** 10.1186/s13018-022-02960-6

**Published:** 2022-02-02

**Authors:** Yifei Gu, Yi Sun, Sohaib Shujaat, Annabel Braem, Constantinus Politis, Reinhilde Jacobs

**Affiliations:** 1 OMFS-IMPATH Research Group, Department of Imaging and Pathology, KU Leuven, Leuven, Belgium; 2 Department of Oral and Maxillofacial Surgery, University Hospitals Leuven, Leuven, Belgium; 3 Department of Materials Engineering, Biomaterials and Tissue Engineering Research Group, KU Leuven, 3000 Leuven, Belgium; 4 Department of Dental Medicine, Karolinska Institutet, Stockholm, Sweden

**Keywords:** Titanium alloy, Ti6Al4V, 3D printing, Animal study, Bone tissue engineering

## Abstract

**Background:**

Titanium and its alloys have been widely employed for bone tissue repair and implant manufacturing. The rapid development of three-dimensional (3D) printing technology has allowed fabrication of porous titanium scaffolds with controllable microstructures, which is considered to be an effective method for promoting rapid bone formation and decreasing bone absorption. The purpose of this systematic review was to evaluate the osteogenic potential of 3D-printed porous Ti6Al4V (Ti64) scaffold for repairing long bone defects in animal models and to investigate the influential factors that might affect its osteogenic capacity.

**Methods:**

Electronic literature search was conducted in the following databases: PubMed, Web of Science, and Embase up to September 2021. The SYRCLE's tool and the modified CAMARADES list were used to assess the risk of bias and methodological quality, respectively. Due to heterogeneity of the selected studies in relation to protocol and outcomes evaluated, a meta-analysis could not be performed.

**Results:**

The initial search revealed 5858 studies. Only 46 animal studies were found to be eligible based on the inclusion criteria. Rabbit was the most commonly utilized animal model. A pore size of around 500–600 µm and porosity of 60–70% were found to be the most ideal parameters for designing the Ti64 scaffold, where both dodecahedron and diamond pores optimally promoted osteogenesis. Histological analysis of the scaffold in a rabbit model revealed that the maximum bone area fraction reached 59.3 ± 8.1% at weeks 8–10. Based on micro-CT assessment, the maximum bone volume fraction was found to be 34.0 ± 6.0% at weeks 12.

**Conclusions:**

Ti64 scaffold might act as a promising medium for providing sufficient mechanical support and a stable environment for new bone formation in long bone defects.

*Trail registration* The study protocol was registered in the PROSPERO database under the number CRD42020194100.

**Supplementary Information:**

The online version contains supplementary material available at 10.1186/s13018-022-02960-6.

## Background

Since the early 1970s, titanium and its alloys have been widely incorporated in the biomedical field for manufacturing implants and repairing bone defects. These alloys offer a lower Young’s modulus compared to other materials, such as stainless steel, cobalt–chromium alloys and tantalum. Nevertheless, an elastic modulus mismatch still exists between titanium and bone tissue which could lead to bone atrophy, fracture, osteoporosis and early implant failure due to the stress shielding effect. To overcome this limitation, porous titanium scaffolds have been designed for reducing the modulus and mimicking the strength of natural bone, thereby allowing prevention of the stress shielding [1, 2] and promotion of scaffold fixation into the surrounding tissues [3, 4]. The interconnected pores of the scaffold also offer a shorter healing time with an improved vascularization and exchange of nutrients. At present, porous titanium scaffolds have been employed in the manufacturing of three-dimensional (3D)-printed implants such as artificial lumbar fusion cages and acetabular joints [5–8]. These implants offer high compressive strength, bone-like elastic modulus, and promote long-term bone ingrowth. Furthermore, they have an optimal wear resistance for resisting scratches, cracks, and peeling [9]. These characteristics make porous titanium-based implants an excellent candidate for repairing bone defects.

Currently, porous titanium-6 aluminium-4 vanadium (Ti6Al4V) scaffold is one of the most commonly used materials for manufacturing load-bearing implants and repairing bone defects owing to its superior mechanical properties and osseointegration compared to commercially pure titanium and other alloys [10]. Many conventional material processing methods have been employed for fabricating porous Ti6Al4V scaffolds, which include sintering [11], solid-state foaming [12], and polymeric sponge replication [13]. In comparison, recent introduction of additive manufacturing (AM) technology and processes, such as selective laser melting (SLM) and electron beam melting (EBM), has allowed the fabrication of customizable 3D-printed porous Ti6Al4V (Ti64) scaffolds which offer predictable and predetermined unit cells. Both the aforementioned technologies belong to the powder bed fusion category, where the heat generated from an energy source (SLM: fiber laser, EBM: electronic beam) is used to selectively melt and fuse powder layer by layer based on the computer-aided design (CAD) model. When one layer of powder has been selectively melted, the build platform is lowered to a predetermined distance and the next layer is deposited. The process is repeated with each successive layer until the desired part is entirely constructed [14].

These 3D-printed porous scaffolds have the potential to replace other bone graft substitutes (allograft, autograft) for treating long bone defects, which are prone to certain risks such as restricted blood supply, disease transmission, and high morbidity rate. The key to reducing these risks is to utilize a porous Ti64 scaffold with interconnected pores having sufficient pore size (> 100 μm), which has the ability to promote cell proliferation and migration, as well as allow generation of new bone and capillaries [15]. Additionally, these scaffolds have a high coefficient of friction against cancellous bone (*μ* = 1.09), which ensures a stable environment for new bone formation [16]. At an early stage following implantation, porous scaffolds also provide mechanical support to the damaged hard tissue [17].

Although the advantages of Ti64 scaffold have been well documented, it is biologically inert and lacks osteoinductivity. Consequently, it allows new bone formation only from the edges where it is in contact with the pre-existing bone, which leads to a delayed complete fill-up of the defect. Ideally, the new bone should start forming at the center of the scaffold. Many studies have attempted to improve the osteogenic effect and fixation of Ti64 scaffold to the surrounding bone for increasing its long-term success rate, which include surface modification techniques (etching, nano-structuring, coating) and addition of growth factors [18]. However, clinical trials involving the implantation of a functional Ti64 porous scaffold are still rare, owing to the technique sensitivity and high costs.

Animal models are the most effective method for confirming the osteogenic potential of these modified functional Ti64 scaffolds by investigating the macroscopic and microscopic changes of the bone environment during osseointegration [19]. While testing the modified Ti64’s osteogenic performance, it is common to use pristine Ti64 scaffold as a control group [4, 20–22], which allows exclusion of any inherent impact from other scaffold modifying materials. However, no data exist confirming the osteogenic properties of a pristine Ti64 scaffold, which in turn could impact the testing process with biased outcomes. It is also crucial to design the experiments more efficiently. For instance, if an experiment time period is set for too long, then both the control and experimental group might show equally distributed bone formation due to overgrowth, making it difficult to analyze the osteogenic differences between both groups without any discernible contrast. Furthermore, there is still room for improvement in AM technology for reducing excessive residual stress and surface roughness of the scaffold. Therefore, it is necessary to review the state of existing Ti64 scaffold’s manufacturing and preclinical testing to better understand its osteogenic potential and yield more effective strategies for improving its clinical applicability.

**Fig. 1:**
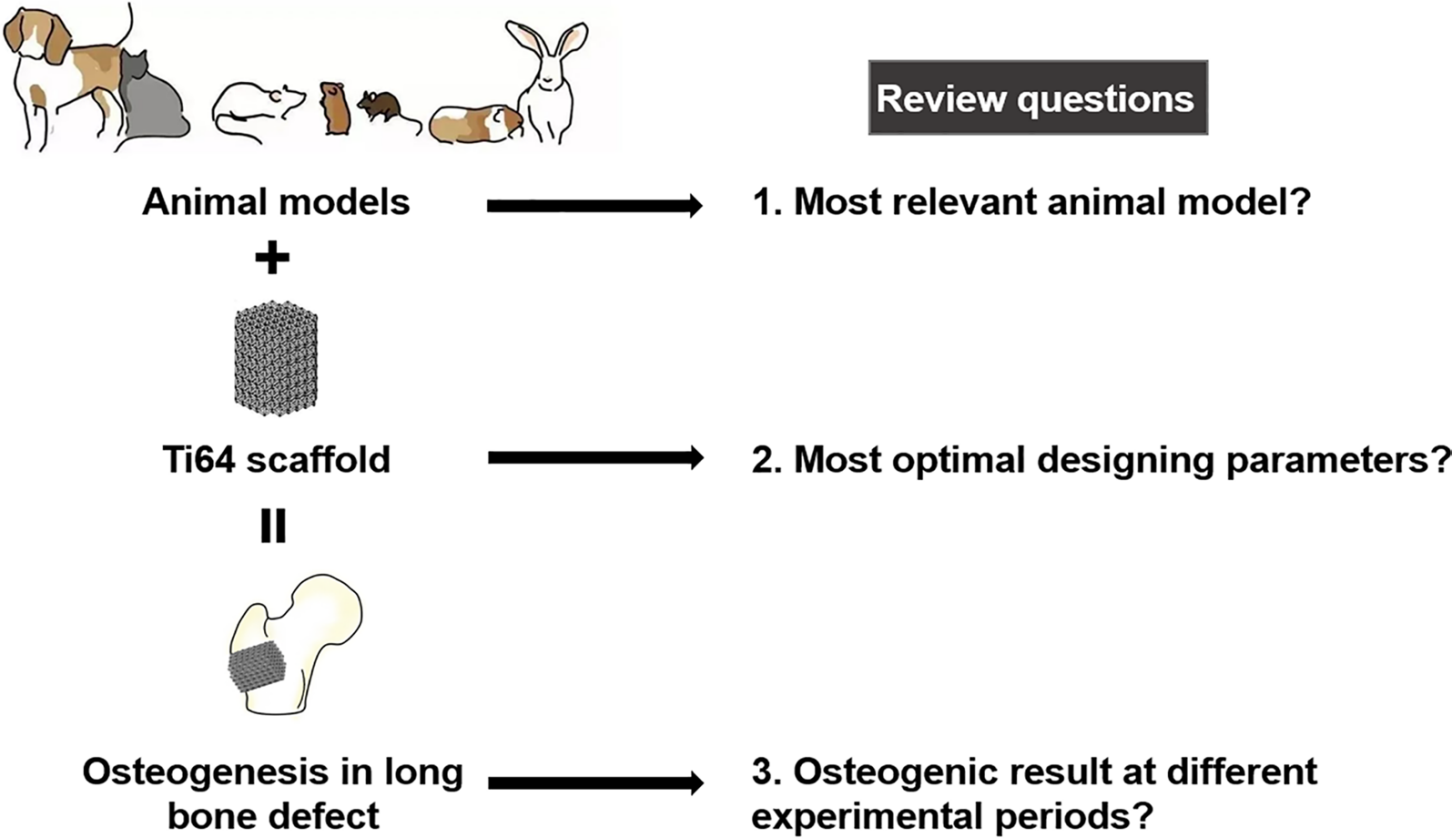
Scope of the systematic review

The following systematic review aimed to report the current evidence related to the application of Ti64 scaffold for repairing long bone defects in animal models and to investigate the potential influential factors that might affect its osteogenic ability. The scope of this systematic review is shown in Fig. 1.

## Methods

The study protocol was registered in the PROSPERO database under the number CRD42020194100. The systematic review followed the Preferred Reporting Items for Systematic Reviews and Meta-Analyses (PRISMA) guidelines. Two researchers (GYF, SY) searched the electronic databases of PubMed, Embase, and Web of Science for relevant studies published till September, 2021. Search keywords were ("Bone/bone regeneration/bone reconstruction") AND ("Titanium alloy") AND ("3D printing"). The detailed search strings are presented in Additional file 1. Grey literature and references within the selected studies were also screened. Identified studies were imported into Endnote online software (Thomson Reuters, Philadelphia, PA, USA) for removing duplicates.

Table 1 describes the inclusion and exclusion criteria. All animal studies which reported on the application of Ti64 scaffold for long bone defect repair were included. “Long bone” was defined as a bone consisting of a tubular shaft (diaphysis) and two extremities (epiphyses), and “Ti64 scaffold” referred to a 3D-printed structure with a network of fully interconnected pores [23].

**Table 1:** Inclusion and exclusion criteria used in this study

Inclusion criteria	Exclusion criteria
All in vivo studies which reported on the application of Ti64 scaffold for long bone defect repair	1. Non-English papers
	2. Descriptive studies, in vitro studies, and clinical trials
	3. Studies that used materials other than Ti6Al4V
	4. Partially porous Ti64 implant or Ti64 implant with only a textured surface layer
	5. Animal models with comorbidities (hypertension, diabetes, osteoporosis, etc.)
	6. Not a long bone defect (defect in cranial bone, jaw bone, etc.)

Two researchers (GYF, SY) independently screened the relevant articles based on the titles and abstracts and then read the full text of the included studies. Any disagreement was resolved through consensus. If an agreement could not be reached, a third researcher (RJ) was consulted. Risk of bias was assessed according to the SYRCLE's tool [24], and the CAMARADES list (www.camarades.info) was used for determining the methodological quality of the included articles.

The extracted data included scaffold characteristics (scaffold size and shape, fabrication method, pore size, porosity), study characteristics (animal model, implantation time, bone defect), and the reported osteogenic outcomes (bone area, bone volume).

The PICO (Population, Intervention, Comparison, and Outcome) criteria were as follows [25]:

- ***Population*:** animals with long bone defect.
- ***Intervention/exposure*:** application of Ti64 scaffold for repairing long bone defect.
- ***Comparison*:** not applicable.
- ***Outcome*:** quantitative assessment of the new bone tissue formation.

## Results

**Fig. 2:**
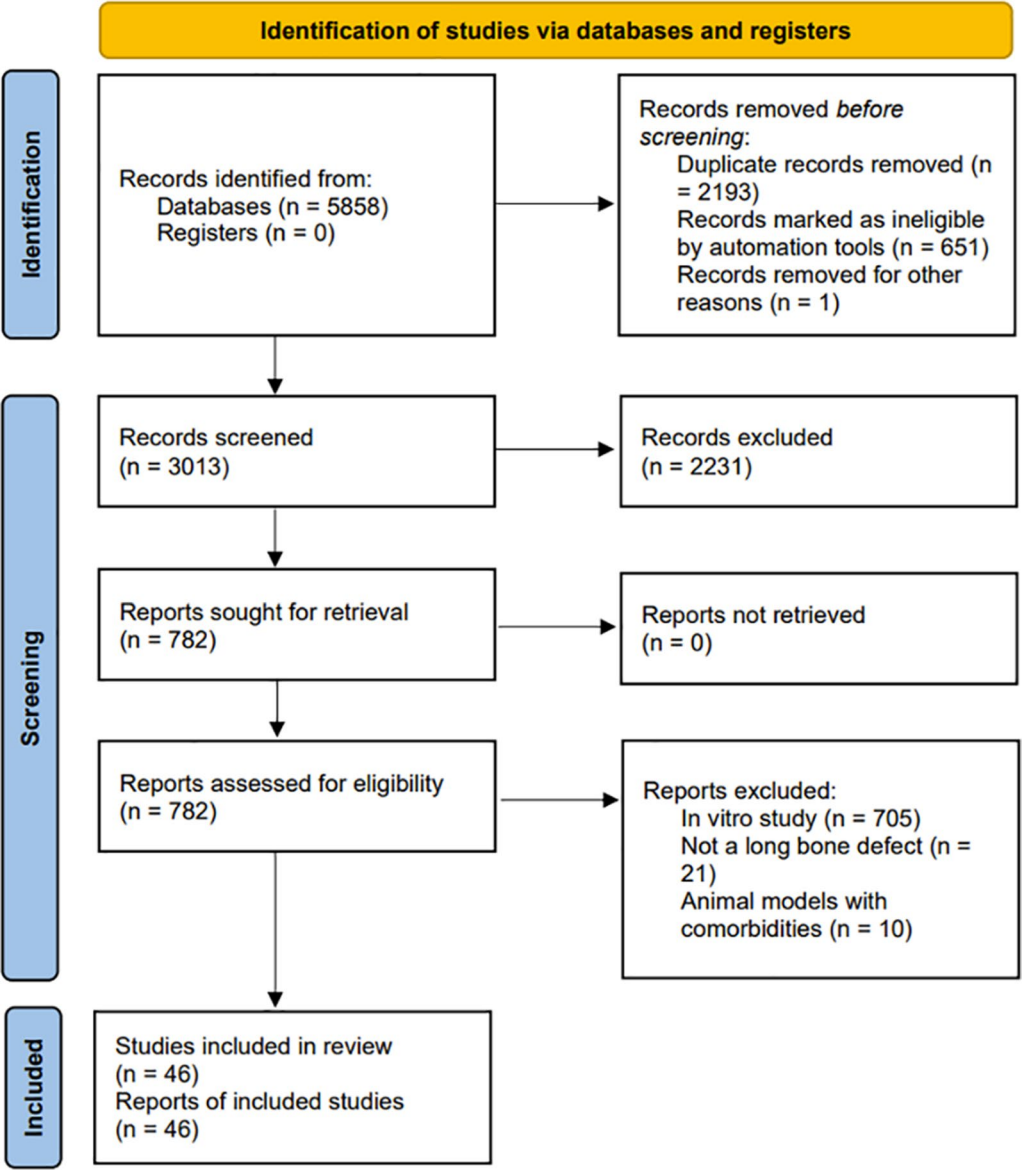
Screening flow diagram based on the PRISMA guidelines

**Fig. 3:**
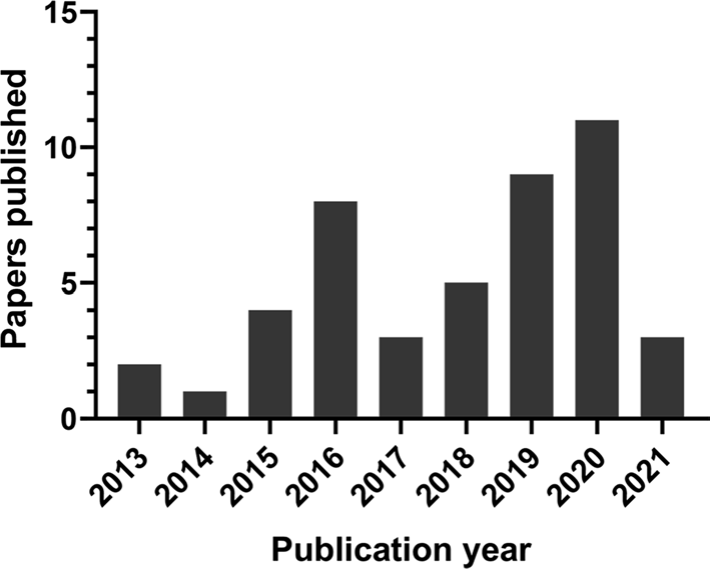
Relationship between the year of publication and number of included articles

Figure 2 illustrates the screening flowchart based on PRISMA guidelines, and the PRISMA checklist can be found in Additional file 2. The search strategy retrieved 5858 articles. Following removal of duplicates, title and abstract screening, and full-text reading, 46 studies were eligible to be included in the review ranging from year 2013 till 2021, with the majority articles being published in 2020 (Fig. 3).

### Quality of the included studies

#### Risk of bias

**Fig. 4:**
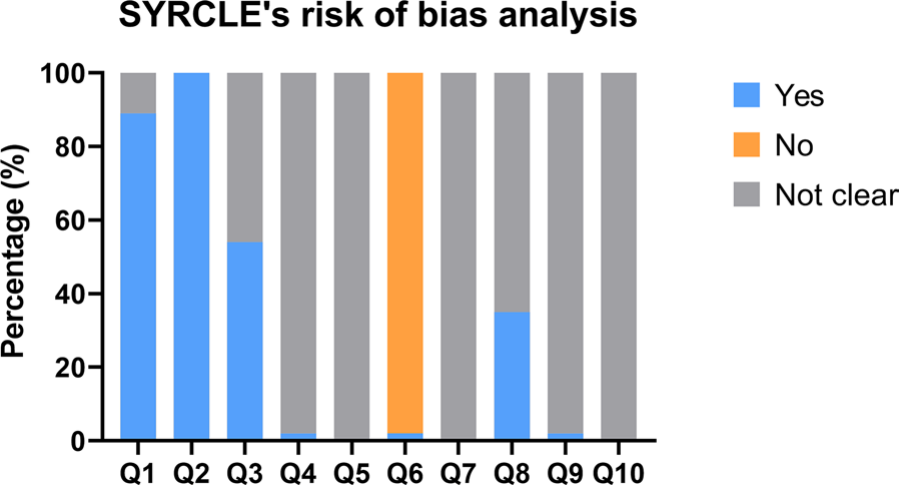
Results of SYRCLE's risk of bias analysis

Figure 4 illustrates the findings of the SYRCLE's risk of bias analysis. The allocation sequence of animals was adequately generated in 41 studies (89%), and the baseline characteristics of the experimental animals were similar in all studies. No study mentioned whether the allocation was adequately concealed. Only one study stated that the animals were housed randomly. There was no explicit description of blind intervention or outcome evaluation. Most studies (45 articles, 98%) did not select animals randomly for outcome assessment due to the high cost of animals. Additionally, many other items in the questionnaire were rated as "unclear," implying that reporting of these animal studies (mainly experimental designs) should be improved.

#### Methodological quality

**Fig. 5:**
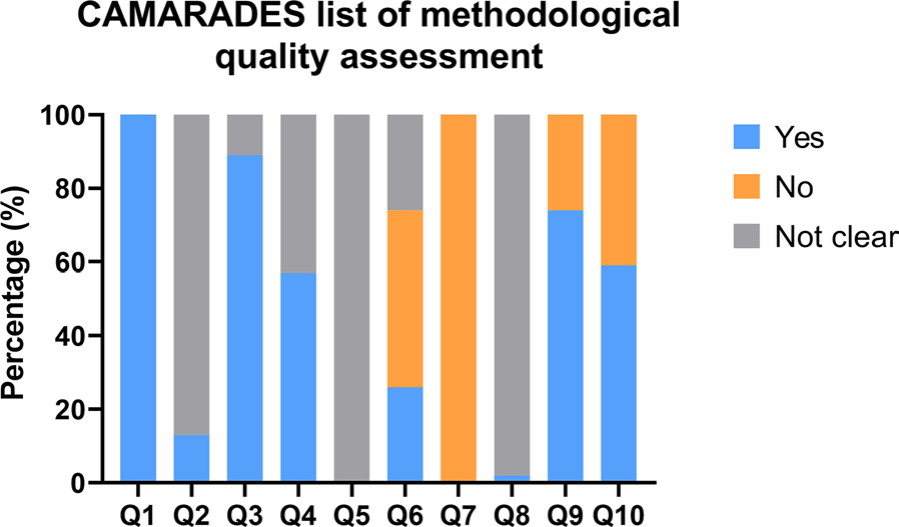
Results of CAMARADES list of methodological quality assessment

Figure 5 illustrates the CAMARADES checklist-assisted methodological quality assessment. The outcomes of animal allocation, allocation concealment, and blind operation assessment were similar to those mentioned in "risk of bias" assessment. Animals in all the studies were healthy, and a neuroprotective anesthetic was administered in some studies (12 articles, 26%). Only one article mentioned the sample size calculation method. Most studies (34 articles, 74%) clarified the adherence to relevant operating guidelines during animal experiments. Furthermore, the rest of the items were rated as "unclear" which matched the description as in "risk of bias" assessment.

### Characteristics of the included studies

#### Scaffold design and animal study setup

**Fig. 6:**
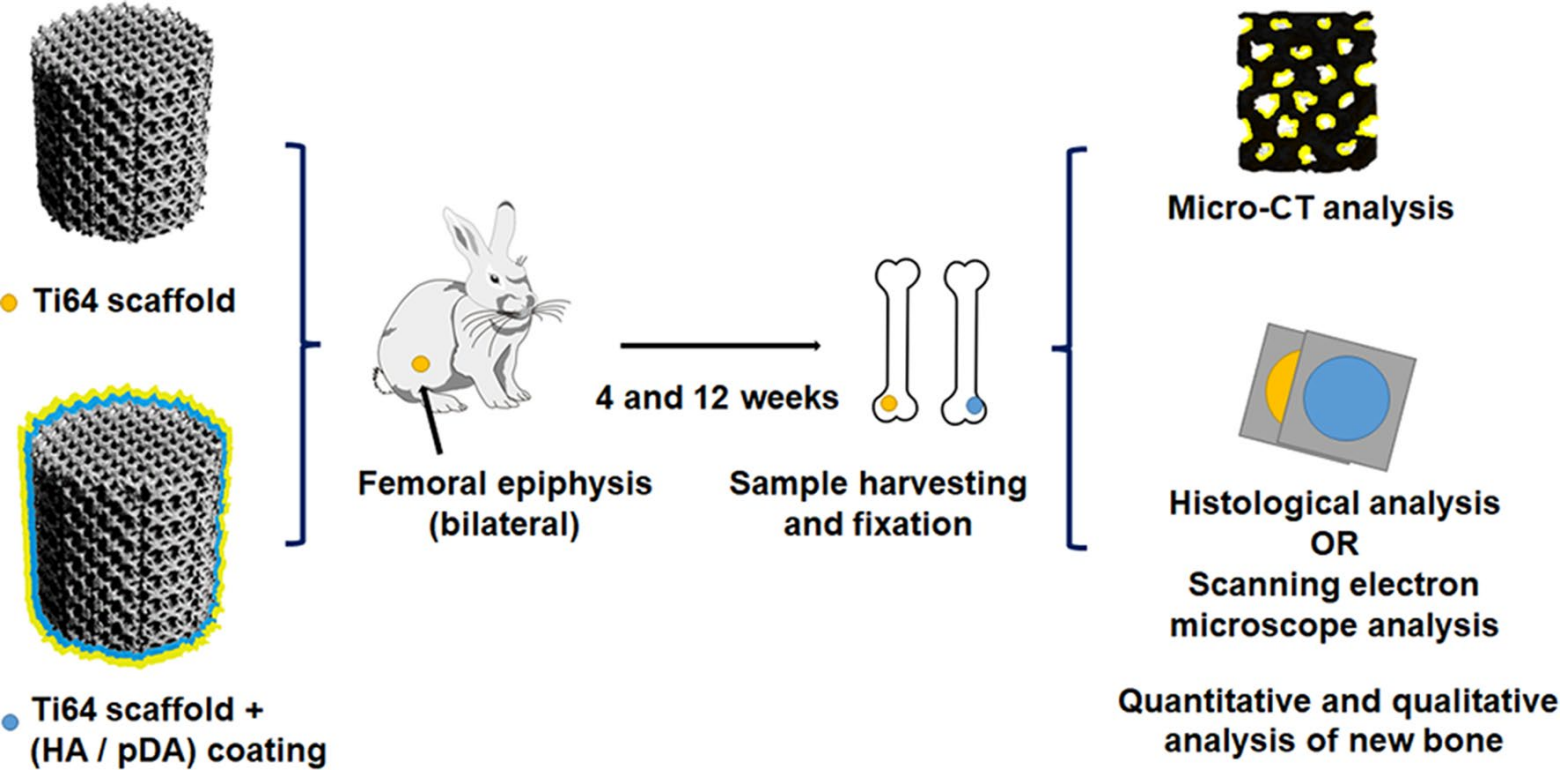
Schematic workflow of a typical animal experiment (Li et al. [41])

Table 2 summarizes the design of the included articles, and Fig. 6 illustrates a schematic workflow of a typical animal experiment. If an author’s last name was the same in different articles, a number was used following the last name to distinguish the authors.

**Table 2:** List of Ti64 scaffolds and animal study designs

Author, date	Fabrication method	Scaffold shape and size (*∅* * *h*, in mm)	Strut size (µm)	Pore size (µm)	Porosity (%)	Unit cell	Modifications to improve biocompatibility	Animal model	Bone defect	Implantation time (weeks)
Arabnejad et al. 2016 [30]	SLM	C(5 * 10)	200–400	438–772	56–70	Tetrahedron Octahedron	/	Dog	Femur	4 and 8
Bandyopadhyay et al. 2019 [4]	SLM	C(3 * 5)	/	60–700	25	/	Anodization treatment	Rat	F-epi	12
Chen et al. 2019 [31]	SLM	C(6 * 6)	300	600	70	Sphere	/	Dog	Tibia	4 and 12
Chen et al. 2020 [32]	SLM	C(3 * 4)	/	500–700	60–70	Octahedron	/	Rat	F-epi	4 and 12
Crovace et al. 2020 [33]	EBM	C(12 * 400)	/	3600	90	Gyroid	/	Sheep	Tibia(s)	48
Fan et al. 2020 [34]	EBM	C(5 * 13)	382–383	659–663	70–71	/	Barium titanate coating	Rabbit	Radius(s)	6 and 12
Gilev et al. 2019 [35]	SLM	Cubic	/	/	/	/	/	Rabbit	Tibia	1,2,6,12 and 48
Guo et al. 2020 [36]	SLM	C(5 * 10)	/	340–360	70–75	/	Titanium copper/titanium copper nitride multilayer coating	Rabbit	F-epi	4, 8 and 12
Guo et al. 2018 [22]	SLM	C(5 * 10)	/	316	74	Cubic	/	Rabbit	F-epi	4, 8 and 12
Han et al. 2016 [37]	EBM	C(5 * 5)	400	600–800	55–67	Cubic	Anodization treatment and strontium incorporation	Rabbit	F-epi	4 and 12
Hara et al. 2016 [16]	EBM	C(5 * 12)	/	501–933	65–70	Diamond	/	Rabbit	Femur	4 and 12
Huang et al. 2017 [20]	EBM	C(10 * 20)	238	514	69	Diamond	HA coating	Goat	F-epi	8 and 16
Kelly et al. 2021 [38]	SLM	C(4.5 * 8)	/	739–1076	70	TPMS-based cell	/	Rat	Femur(s)	12
Koolen et al. 2020 [39]	SLS	C(5 * 6)	211	244	79	Rhombic dodecahedron	AlAcH treatment	Rat	Femur(s)	11
Li et al. 2019 [40]	SLM	C(5 * 6)	/	400	45	Cubic	Polydopamine coating	Rabbit	F-epi	5
Li et al. 2019 [29]	SLM	C(8 * 10)	/	100–700	50	TPMS-based cell	/	Pig	Tibia	5
Li et al. 2016 [3]	EBM	C(10 * 30)	200	315	34	Diamond	/	Goat	Metatarsus(s)	12, 24 and 48
Li et al. 2015 [41]	EBM	C(5 * 10)	/	710	68	/	Polydopamine-assisted hydroxyapatite coating	Rabbit	F-epi	4 and 12
Liu et al. 2016 [42]	EBM	C(5 * 16)	400	640	76	/	Addition of simvastatin and hydrogel	Rabbit	Tibia	4 and 8
Liu et al. 2020 [43]	SLM	C(10 * 10)	/	600	65	/	/	Rabbit	F-epi	4, 6, 8, 10 and 12
Luan et al. 2019 [44]	EBM	C(5 * 4)	/	334–402	55–78	/	/	Rabbit	Femur	12
Lv et al. 2015 [45]	EBM	C(5 * 6)	/	640	/	Hexagonal	Addition of bone morphogenetic protein-2 (BMP-2), vascular endothelial growth factor and fibringel	Rabbit	F-epi	4
Lyu et al. 2020 [46]	/	C(2 * 5)	320	650	70	/	/	Rabbit	F-epi	12
Ma et al. 2018 [47]	SLM	C(5 * 6)	/	400	76	/	Addition of mineralized collagen	Rabbit	Radius(s)	4 and 12
Ma et al. 2021 [48]	SLM	C(15 * 20)	/	400	76	/	Addition of gelatin methacrylate	Rabbit	Radius(s)	4 and 12
Mumith et al. 2020 [49]	SLS	C(8 * 14.5)	300–750	700–1500	70–75	/	HA coating, silicon-substituted or strontium-substituted HA coating	Sheep	F-epi	6
Palmquist et al. 2017 [50]	EBM	C(5.2 * 5)	350	550	70	Diamond	/	Sheep	Femur and tibia	24
Palmquist et al. 2013 [51]	EBM	C(5.2 * 7)	500–1000	500–700	65–70	/	/	Sheep	F-epi	26
Ragone et al. 2020 [26]	SLM	C(6.2 * 11)	200	450–1200	75–90	Irregular-shaped	/	Sheep	Femur and tibia	6, 10 and 14
Ran et al. 2018 [52]	SLM	C(4 * 13)	300–400	401–801	/	Circle	/	Rabbit	F-epi	4 and 12
Shah et al. 2016 [53]	EBM	C(5.2 * 7)	583	/	63	/	/	Sheep	F-epi	24
Shah et al. 2016 [54]	EBM	C(5.2 * 5)	341	470–545	70	Diamond	/	Sheep	F-epi	26
Song et al. 2019 [55]	SLM	C(3 * 6)	200–400	/	65–86	Diamond	HA coating	Rabbit	Femur	12 and 24
Tanzer et al. 2019 [19]	SLM	C(5.2 * 10)	/	450	50–65	Irregular-shaped	HA coating	Dog	Femur	/
Tsai et al. 2019 [56]	SLM	C(6.5 * 10)	/	350	/	Cubic	Magnesium–calcium silicate and chitosan coating	Rabbit	Femur	6
van der Stok et al. 2015 [57]	SLM	Femur-shape	165	577	85	Rhombic dodecahedron	AlAcH surface treatment and addition of BMP-2 and fibrin gel	Rat	Femur(s)	12
van der Stok et al. 2015 [58]	SLM	Femur-shape	120	500	88	Rhombic dodecahedron	AlAcH surface treatment and osteostatin coating	Rat	Femur(s)	12
van der Stok et al. 2013 [17]	SLM	Femur-shape	120–230	490	68–88	Rhombic dodecahedron	AlAcH treatment	Rat	Femur(s)	4,8 and 12
Wang et al. 2018 [27]	SLM	C(4.8 * 8)	410–449	427–458	61–66	Diamond Tetrahedron	/	Rabbit	Femur	4 and 8
Wang et al. 2018 [59]	/	C(8 * 10)	/	200	/	/	Strontium ion incorporated zeolite coating	Rabbit	F-epi	4
Xiu et al. 2017 [60]	EBM	C(6 * 5)	400	640	73	Rhombic dodecahedron	Hybrid micro-arc oxidation and hydrothermal treatment	Rabbit	F-epi	8
Xiu et al. 2017 [61]	EBM	C(6 * 5)	400	640	73	Rhombic dodecahedron	Hybrid micro-arc oxidation	Rabbit	F-epi	8
Yavari et al. 2014 [62]	SLM	Femur-shape	160–180	577–596	85–89	Rhombic dodecahedron	Acid–alkali treatment, AlAcH treatment and anodizing-heat treatment	Rat	Femur(s)	4, 8 and 12
Yu et al. 2020 [28]	SLM	Cone-shape	200	650	90	Rhombic dodecahedron	/	Rabbit	Femur	4 and 8
Zhang et al. 2021 [63]	SLM	C(5 * 10)	300	/	68	Diamond	Bioactive glass and mesoporous bioactive glass coating	Rabbit	F-epi	6 and 9
Zhong et al. 2020 [64]	SLM	C(6 * 10)	/	/	/	/	/	Rabbit	F-epi	6 and 12

*C: cylinder; HA: hydroxyapatite; AlAcH: alkali-acid-heat; F-epi: femoral epiphysis; s: segmental bone defect; wk: weeks; m: months

*Data are all average values, and parameters were reserved for integers

*If multiple Ti64 scaffold design parameters have been applied in a single article, the parameters are expressed in ranges

A total of 29 studies (63%) mentioned the type of Ti6Al4V powder for 3D printing, with particle sizes ranging from 15 to 100 µm. In 16 studies, Grade 23 Ti6Al4V powder was incorporated. The 3D printing technique was mentioned in 44 studies (96%), which included; SLM, (26 studies, 59%), EBM (16 studies, 36%), and selective laser sintering (SLS) (2 studies, 5%). Post-processing of the Ti64 scaffold was mentioned in 30 studies (65%), which involved removal of excess powder with either ultrasonic cleaning, sandblasting or acid treatment.

The design of choice for the scaffold was cylindrical in most studies (40 studies, 87%) with the size variation depending on the animal model and reconstruction method. The strut size varied between 60 and 3600 µm, mostly ranging between 200 and 400 µm (20 studies, 77%). The reported pore size was 100–1500 µm, with the size of 500–700 µm in the majority of studies (23 studies, 56%), followed by 300–499 µm (16 studies, 39%). The porosity of Ti64 scaffold ranged from 25 to 90% in 40 studies; however, 60–70% was applied in most of the studies (22 studies, 55%). Various pore shapes could be observed depending on the unit cell for designing the lattice structure, where some studies reported on more than one pore shape. Rhombic dodecahedron (8 studies, 27%) and diamond (8 studies, 27%) were the most commonly applied unit cell shapes. Other shapes included octahedron, tetrahedron, cube, spiral tetrahedron, hexagon, and triply periodic minimal surface (TPMS)-based cells. Most studies (42 studies, 91%) adopted a regularly arranged lattice structure, while others used a randomly generated or arranged irregular pore structure [19, 26–28].

In this review, the randomly distributed pore structures were divided into the following categories: 1. Pore structure designed based on the Voronoi tessellation method [26]; 2. Pore structure designed using TPMS model [29]; 3. Randomly generated pore structure with varying pore sizes and shapes [19]; 4. Regular-shaped and regular-sized pore structure with randomly arranged pores [27, 28].

The mechanical strength of the Ti64 scaffold was reported in 15 studies, ranging from 14 to 606 MPa, where 10 studies (66%) described its strength between 30 and 200 MPa. The elastic modulus varied from 0.32 to 7.56 GPa, with the majority falling between 0 and 3 GPa (12 studies, 63%).

Rabbit was the most commonly utilized animal model (25 articles, 54%). Other animal models included rat, sheep, dog, goat and pig. Femur was the preferred implantation site in the majority of studies (36 studies, 78%, femoral diaphysis: *n* = 15, femoral epiphyses: *n* = 21), while other sites included tibia and radius. Non-segmental bone defects accounted for most cases (35 studies, 76%). The experiment duration ranged from 4 weeks to 12 months, and the follow-up time period for most articles was less than 12 weeks (36 studies, 78%). Additionally, half of the studies assessed outcomes at two or more time-points (26 papers, 57%).

#### Bone tissue outcomes

Quantitative evaluation of the new bone tissue formation was performed by either histomorphometric analysis, scanning electron microscopy (SEM) or 3D micro-CT assessment. The most commonly assessed two-dimensional (2D) and 3D parameters involved bone area fraction (BA/TA, new bone area over scaffold pore area) and bone volume fraction (BV/TV, new bone volume over scaffold pore volume), respectively. An overview of the outcomes is summarized in Tables 3 and 4. The addition of coating material, surface treatment, or other additives might alter the osteogenic performance of a Ti64 scaffold, hence, these conditions were not considered. Furthermore, segmental bone defects were excluded from the BA/TA and BV/TV analyses, as in such cases new bone only grows inward from both scaffold ends which results in a relatively low bone ingrowth compared to a single bone defect where most of the scaffold surface is covered by bone. Due to the heterogeneity in relation to study protocols and reported outcomes among the included studies, no meta-analysis could be performed.

**Table 3:** Summary of reported BA/TA for pristine Ti64 scaffolds in the reviewed studies

Author, date	Animal and bone defect	Group	BA/TA (%)			
			4–6 weeks	8–10 weeks	12–14 weeks	24–26 weeks
Arabnejad et al. 2016 [30]	Beagle dog, femur	Tetrahedron cell	28.6 ± 11.6	41.3 ± 4.3	/	/
		Octet truss cell	35.5 ± 1.9	56.9 ± 4.		
Chen et al. 2019 [31]	Beagle dog, tibia	/	11.9 ± 2.2	/	15.9 ± 4.9	/
Guo et al. 2020 [36]	Rabbit, f-epi	/	11.6 ± 1.9	12.2 ± 2.0	24.1 ± 3.0	/
Guo et al. 2018 [22]	Rabbit, f-epi	/	13.2 ± 2.7	35.6 ± 2.7	55.9 ± 2.0	/
Han et al. 2016 [37]	Rabbit, f-epi	Pore size 600 μm	2.3 ± 0.4	/	3.5 ± 0.5	/
		Pore size 800 μm	1.5 ± 0.1	/	2.4 ± 0.4	/
Hara et al. 2016 [16]	Rabbit, femur	Pore size 500 μm	34.9 ± 6.8	/	50.1 ± 8.3	/
		Pore size 640 μm	37.0 ± 5.0	/	50.9 ± 6.7	/
		Pore size 800 μm	27.2 ± 7.2	/	51.6 ± 6.4	/
		Pore size 1000 μm	34.7 ± 8.4	/	35.1 ± 2.7	/
Li et al. 2015 [41]	Rabbit, f-epi	/	5.8 ± 2.2	/	12.2 ± 2.2	/
Lv et al. 2015 [45]	Rabbit, f-epi	/	7.8 ± 2.8	/	/	/
Palmquist et al. 2017 [50]	Sheep, femur and tibia	Scaffold in femur	/	/	/	Central: 26.5 ± 9.2 Peripheral: 57.2 ± 10.9
		Scaffold in tibia	/	/	/	Central: 45.6 ± 19.5 Peripheral: 8.0 ± 10.4
Palmquist et al. 2017 [51]	Sheep, f-epi	/	/	/	/	44.7 ± 4.4
Ragone et al. 2020 [26]	Sheep, femur and tibia	/	Cortical: 75.0 ± 13.5 Cancellous: 27.0 ± 15.0	Cortical: 82.0 ± 5.0 Cancellous: 36.0 ± 10.5	Cortical: 82.0 ± 9.0 Cancellous: 51.0 ± 14.0	/
Shah et al. 2016 [54]	Sheep, f-epi	/	/	/	/	Central: 32.9 ± 4.8 Peripheral: 60.0 ± 4.6
Song et al. 2019 [55]	Rabbit, femur	/	/	/	6.8 ± 2.9	35.6 ± 5.3
Tanzer et al. 2019 [19]	Beagle dog, femur	/	41.5 ± 8.2	/	64.4 ± 2.8	/
Tsai et al. 2019 [56]	Rabbit, femur	/	2.5 ± 0.8	/	/	/
Wang et al. 2018 [27]	Rabbit, femur	Diamond cell (r)	34.0 ± 5.9	36.3 ± 1.0	/	/
		Diamond cell (ir)	33.7 ± 5.0	36.8 ± 2.3	/	/
		Diamond cell (g)	30.2 ± 3.3	32.3 ± 4.9	/	/
		Tetrahedron cell	20.5 ± 3.0	24.3 ± 1.9	/	/
Xiu et al. 2017 [60]	Rabbit, f-epi	/	/	8.2 ± 2.3	/	/
Xiu et al. 2017 [61]	Rabbit, f-epi	/	/	10.8 ± 3.4	/	/
Yu et al. 2020 [28]	Rabbit, femur	/	46.3 ± 13.7	59.3 ± 8.1	/	/

*F-epi: femoral epiphysis; r: regularly distributed pores; ir: irregularly distributed pores; g: gradient distributed pores

*The data are all represented as means ± standard deviations, and reserved for one decimal point

**Table 4:** Summary of reported BV/TV for pristine Ti64 scaffolds in the reviewed studies

Author, date	Animal and bone defect	Group	BV/TV (%)			
			4–6 weeks	8–10 weeks	12 weeks	16 weeks
Chen et al. 2020 [32]	Rat, f-epi	Porosity 60%, pore size 500 μm	/	/	23.4 ± 1.6	/
		Porosity 60%, pore size 600 μm	/	/	21.0 ± 2.1	/
		Porosity 60%, pore size 700 μm	/	/	12.8 ± 2.1	/
		Porosity 70%, pore size 500 μm	/	/	23.2 ± 1.8	/
		Porosity 70%, pore size 600 μm	/	/	22.3 ± 1.0	/
		Porosity 70%, pore size 700 μm	/	/	18.3 ± 1.4	/
Guo et al. 2020 [36]	Rabbit, f-epi	/	11.6 ± 1.8	17.1 ± 1.6	25.5 ± 2.6	/
Han et al. 2016 [37]	Rabbit, f-epi	Pore size 600 μm	8.4 ± 1.3	/	16.2 ± 3.6	/
		Pore size 800 μm	4.3 ± 1.0	/	8.6 ± 2.7	/
Huang et al. 2017 [20]	Goat, f-epi	/	/	5.1 ± 1.8	/	6.3 ± 2.2
Li et al. 2019 [40]	Rabbit, f-epi	/	13.7	/	/	/
Li et al. 2019 [29]	Pig, tibia	Pore size 300-500 μm	12.7 ± 3.6	/	/	/
		Pore size 200-600 μm	12.0 ± 3.6	/	/	/
		Pore size 100-700 μm	12.8 ± 3.9	/	/	/
Li et al. 2015 [41]	Rabbit, f-epi	/	5.9 ± 2.2	/	11.0 ± 2.6	/
Liu et al. 2016 [42]	Rabbit, tibia	/	26.7 ± 1.0	28.9 ± 1.4	/	/
Luan et al. 2019 [44]	Rabbit, femur	Porosity 55%, pore size 334 μm	/	/	21.4 ± 2.2	/
		Porosity 65%, pore size 383 μm	/	/	24.6 ± 2.0	/
		Porosity 78%, pore size 400 μm	/	/	26.7 ± 0.9	/
Lyu et al. 2020 [46]	Rabbit, f-epi	/	/	/	34.0 ± 6.0	/
Wang et al. 2018 [59]	Rabbit, f-epi	/	6.0 ± 0.2	/	/	/
Yu et al. 2020 [28]	Rabbit, femur	/	27.3 ± 8.4	29.8 ± 2.2	/	/
Zhang et al. 2021 [63]	Rabbit, f-epi	/	13.4 ± 1.0	16.6 ± 2.18	/	/
Zhong et al. 2020 [64]	Rabbit, f-epi	/	13.9 ± 1.5	/	16.0 ± 1.3	/

*F-epi: femoral epiphysis

*The data are all represented as means ± standard deviations, and reserved for one decimal point

1. BA/TA analysis (Table 3)

The majority of BA/TA data were obtained from rabbit models (12/19 articles, 63%), where scaffold was implanted either in the region of femoral diaphysis (5 articles) or femoral epiphysis (7 articles). The BA/TA in rabbit models ranged from 1.5 ± 0.1% to 46.3 ± 13.7% at weeks 4–6, 8.2 ± 2.3% to 59.3 ± 8.1% at weeks 8–10, 2.4 ± 0.4% to 51.6 ± 6.4% at weeks 12–14, and 35.6 ± 5.3% at weeks 24–26 (only reported by Song et al. [55]). The maximum BA/TA at weeks 4 and 8 was only reported by Yu et al. [28]. Additionally, Hara et al. [16] observed that the depth of new bone ingrowth at week 12 exceeded 1.5 mm in a rabbit model.

Four studies provided BA/TA data in sheep models, and only Palmquist et al. [51] found that BA/TA reached 44.7 ± 4.4% in femoral epiphysis at week 26. The remaining three studies calculated the BA/TA at different scaffold regions (central part vs. peripheral part; cortical bone vs. cancellous bone). The new bone formation was significantly higher in the cortical bone and at the peripheral region of the Ti64 scaffold compared to cancellous bone and central area [26, 50, 54].

Three studies assessed BA/TA at the region of femoral or tibial diaphysis in beagle dogs. It was found to be within the range of 11.9 ± 2.2% to 41.5 ± 8.2% at weeks 4–6, 41.3 ± 4.3% to 56.9 ± 4.0% at weeks 8–10 (only reported by Arabnejad et al. [30]), and 64.4 ± 2.8% at weeks 12–14 (only reported by Tanzer et al. 2019 [19]).

2. BV/TV analysis (Table 4)

As BA/TA is limited to 2D sections, BV/TV based on micro-CT analysis provides a more comprehensive quantification of bone ingrowth. Rabbit model was applied in the majority of studies (11/14 articles, 79%) and the implantation site included femoral epiphysis (8 articles) and femoral diaphysis (3 articles. The BV/TV in rabbits ranged from 4.3 ± 1.0% to 27.3 ± 8.4% at weeks 4–6, 16.6 ± 2.18% to 29.8 ± 2.2% at weeks 8–10, and 8.6 ± 2.7% to 34.0 ± 6.0% at week 12. The maximum BV/TV at weeks 4–6 and weeks 8–10 was only reported by Yu et al. [28]. Furthermore, the maximum BV/TV reached 12.8 ± 3.9 at weeks 4–6 in pigs, 23.4 ± 1.6% at week 12 in rats, and 6.3 ± 2.2% at week 16 in goats.

#### Variables affecting the osteogenic capacity of Ti64 scaffold

From the selected studies and the data presented in Tables 3 and 4, some variables have been summarized below, which might impact the osteogenic ability of a Ti64 scaffold:

*Implantation site:* Ragone et al. [26] found that the new bone formation in the cortical bone region was significantly greater compared to cancellous bone and the osseointegration almost completed after 2 months.

*Implantation time:* The maximum BA/TA values in rabbit models peaked at weeks 8–10. The maximum BV/TV values in rabbit models and the maximum BA/TA values in beagle dog models increased from week 4 till week 12.

*Pore size and porosity*: Hara et al. [16] proposed that a pore size of 500–800 µm was optimal for new bone growth. Similarly, Ran et al. [52] observed that a pore size of 600–800 µm had greater osteogenic ability compared to a size of 400 µm. Furthermore, Chen et al. and Han et al. found that a scaffold with pore size of 500–600 µm has more osteogenic capability compared to 700–800 µm [32, 37]. In terms of porosity, Luan et al. suggested that 78% porosity had a higher osteogenic capacity than 65% and 55% porosity.

*Pore shape*: Within a Ti64 scaffold with high porosity (> 50%), Arabnejad et al. [30] observed that new bone formation in an octagonal shaped pore was more prominent compared to tetrahedral structure. Furthermore, Wang et al. [27] observed a lower bone formation with a tetrahedral pore structure compared to diamond shape.

## Discussion

Over the past few years, animal experimentations for confirming the osteogenic potential of Ti64 scaffolds have gradually gained attention in the biomedical field. Therefore, the following review was conducted to accumulate evidence and report on the osteogenic ability of Ti64 scaffold for repairing long bone defects and to investigate influential factors which might impact its effectiveness. Meta-analysis could not be performed due to the presence of significant heterogeneity among the selected studies related to the scaffold design and size, defect type, and observation time. However, the qualitative evidence synthesis suggested certain critical commonalities and limitations associated within the the methodologies of the included studies for fabricating Ti64 scaffold with optimal osteogenic potential, which could act as a reference guide for future comparative studies.

When considering the animal model for the validation of scaffold, it is essential to understand the bone healing capacity of different animal species. Bone remodeling in small rodents is much faster than larger species [65]. Additionally, rabbits and dogs have a higher bone remodeling rate compared to humans. Therefore, it might be difficult to extrapolate the osteogenic response in these animals for a possible similar response in humans [66]. However, sheep, goat, and pig offer a similar bone remodeling rate to that of humans, making them a better choice for generating osteogenic responses and translating those findings to humans [67, 68].

In terms of the bone healing process, rats are considered less suitable based on the lack of haversian system. However, their bone remodeling is similar to the haversian remodeling in large animals [69]. Thereby, the absence of the haversian system should not be the sole reason for excluding rodents from studies where bone healing assessment is required. Furthermore, the bone healing process of dog, rabbit, sheep, and pig models is remarkably similar to that of humans [70, 71].

The studies using a rat model in this review created segmental bone defects and the scaffold size was very small. We believe that rats should not be considered for assessing the performance of Ti64 scaffolds due to their size limitation and inability to insert multiple implants. Based on the International Organization for Standardization (ISO10993-6:2016(E)), the recommended implant size for biological evaluation in rabbits should be 2 × 6 mm and 4 × 12 mm in larger animals,such as sheep, goat, and dog. The guidelines also recommend an observation time of 1 to 4 weeks’ for assessing short-term outcomes and 12 weeks or more for long-term assessment. Additionally, the long-time follow-up time points for the animal models except rats should be 13, 26, 52, 78, and 104 weeks. However, the findings of the current review revealed that the experimental design in the majority of studies did not follow the international standards and had a follow-up period of less than 12 weeks, hence confirming a lack of evidence related to the standardized long-term outcome evaluation of Ti64 scaffold.

Rabbit was the most commonly applied animal model, and only a few studies assessed the osteogenic capacity of the scaffold with a large animal model such as sheep, goat, dog, and pig. In contrast with large models, small animals are easier to handle, less expensive, and appropriate for screening implant materials before testing in larger models. Unlike small animals, the large animal models have similar bone healing capabilities to that of humans. At the same instance, it should be kept in mind that every large animal model also has its pros and cons. For instance, dogs offer an optimal model for assessing Ti64 scaffold’s effectiveness; however, due to ethical concerns and increased public scrutiny their application in animal research has been declining [72]. An adult sheep has similarities in weight, metabolism, and bone remodeling rates to that of humans and could be considered ideal for testing the scaffold and transferring the findings to a clinical setting [73]. However, researchers tend to use young animals due to financial constraints, which might underestimate the effectiveness of the scaffold as their bones have not fully matured.

In this review, most included studies adopted the femoral condyle defect model or the transcortical defect model. The femoral condyle defect model is most relevant to plastic surgery applications [74]. When there are many groups of scaffolds to be tested on the same animal model at the same time (for example, in the paper of Arabnejad et al. [30], there are four groups of scaffolds to be tested on the same animal model at the same time), the use of transcortical defects is a viable option for achieving greater consistency within the animal. Segmental bone defects were excluded from the review as they are more prone to failure due to bending or breaking of the fixation plate, screw failure, infection, and muscular and neurovascular damage. Additionally, inclusion of segmental defects would have led to bias within the findings of the review, as their mechanical integrity differs from that of a single defect which could negatively impact the osteogenic efficiency and functional recovery of the bone defect.

Based on the findings of the review, Ti64 scaffold induced remarkable osteogenesis in the cortical region compared to cancellous bone. A possible explanation could be the penetration of a lower mechanical stimulus into the cancellous bone surrounding the scaffold [49]. Additionally, the cancellous bone has a randomly distributed pore structure, which cannot be replicated by the regularly distributed simple pattern. In contrast, the randomly distributed pore structures demonstrated higher permeability allowing optimal bone ingrowth [19, 28]. The newly formed bone continues to grow over time with the maximum BA/TA and BV/TV peaking at both weeks 4–6 and weeks 8–10 due to the osteogenic effect of the randomly distributed pores which outperform a simple topological distribution [19].

The osseointegration of Ti64 scaffold is primarily determined by the pore size, structure, porosity, and interconnectivity [19]. The pore size in the included studies ranged from 100 to 900 µm [75]. A large pore size promotes the growth of blood vessels but reduces the mechanical properties and cell adhesion. On the contrary, a small pore size improves cell adhesion and tissue growth; however, the likelihood of pore blockage increases [76]. The findings of the review suggested that the pore sizes ranging from 500 to 600 µm were found to be most optimal for an in vivo fabrication of Ti64 scaffold. Furthermore, an ideal porosity for porous scaffolds should be around 80–90% [76]. The majority of Ti64 scaffolds included in the review had a porosity of 60–70% for ensuring optimal mechanical strength. Furthermore, their compressive strength (33–193 MPa) and elastic modulus (0.02–3 GPa) were equal to that of human cortical bone for avoiding the stress shielding effect [75]. A higher porosity than the aforementioned limit should be avoided to inhibit weakening of the scaffold's mechanical properties.

The pore shape of the Ti64 scaffold varied among studies without any consensus on which shape offered the most optimal outcomes. The most commonly applied shapes were diamond and rhombic dodecahedron. The diamond lattice is isotropic in nature with evenly distributed deformation, which helps to reduce stress concentration. Its structure is closely similar to that of cancellous bone and has a large curvature radius which induces higher tissue amplification [27]. Based on these benefits, a diamond lattice is widely incorporated in porous scaffolds [77]. On the other hand, rhombic dodecahedron lattice has a higher yield stress and is stable under multi-directional compression forces, thereby making it an ideal choice for manufacturing load-bearing implants [28, 78]. However, care should be taken that the dodecahedron shape is designed with only obtuse angles, as acutely angled pores are easily damaged during the melting stage of printing. Other designed pore shapes in the review involved tetrahedral, octahedral, gyroid, and TPMS lattices which are beneficial for improving the scaffold’s strength and rigidity, isotropy, load resistance and surface area, and permeability, respectively [30, 32, 33, 38].

In this review, all Ti64 scaffolds were implanted immediately following the creation of bone defect, which is clinically applicable for procedures where sufficient pre-operative time is available for treatment planning such as lumbar intervertebral fusion and acetabular joint reconstruction. An immediate insertion allows for a reduction in potential complications and cost of the procedure. However, autologous bone graft and bone lengthening with external fixation still remain the standard for treating trauma-related bone defects, where immediate 3D printing is not possible due to a long scaffold designing and printing time.

The review only reported the osteogenic outcomes of unmodified Ti64 scaffold, which could act as a guide for comparison with other porous biomaterials in future studies. However, the impact of scaffold’s surface modification for improving its biological activity and osteogenic capacity cannot be ignored. Surface modification techniques such as anodic oxidation and micro-arc oxidation increase the surface area of the bone in contact with the oxidized scaffold, leading to a quicker and firmer integration of the implant [60, 61]. Hydroxyapatite (HA) coating has also been widely used as a coating agent which demonstrated improved osseointegration and bone ingrowth compared to an uncoated Ti64 scaffold [20, 41]. Furthermore, ion-substituted HA coatings (strontium and silicon substituted HA coatings) also offer an increased bone growth compared to pure HA [49]. Other coatings that have also shown to improve bone ingrowth, wear resistance, and osteogenic capacity of the scaffold include polydopamine coating [40], magnesium–calcium silicate composite coating [56], osteostatin coating [58], Sr-incorporated zeolite coating [59], and titanium-copper/titanium-copper nitride multilayer [36]. Apart from surface modifying techniques and coatings, the Ti64 scaffold has also been tailored with growth factors such as bone morphogenetic protein-2 (BMP-2) and vascular endothelial growth factor (VEGF) for inducing osteogenesis and angiogenesis, respectively [41, 45, 57]. In summary, all the aforementioned factors should be considered when designing a Ti64 scaffold to allow for optimal performance.

Based on the findings of the review, future in vivo studies are warranted using large animal models. Although small animals are acceptable for proving general principles, repeated experiments with larger animal models are necessary for translating the results to humans. Furthermore, as the mechanism of bone regeneration is also dependent on the size of the bone defect, it constitutes a need for creating large sized defects that can only be set in large animal models. Future research should also be conducted with a long-term follow-up period for assessing the precise osteogenic capacity of a Ti64 scaffold. It is also recommended to follow the ISO guidelines for designing experiments and standardized parameters such as BA/TA, BV/TV, and bone-implant-contact should be assessed for quantifying bone ingrowth. These proposed recommendations can greatly reduce data heterogeneity and improve study comparability.

At present, Ti64 scaffold is still a black box for researchers and further exploration is required to unravel its full potential. For instance, a more advanced functional Ti64 scaffold hierarchical design could be fabricated by combining different techniques such as topology optimization, CAD, and minimal surface formulation [9]; Furthermore, surface nano-topography modification could endow Ti64 scaffolds with additional biological functions such as antibacterial properties and enhanced osteogenic differentiation [79]. Another avenue of investigation could be the improvement in the AM processes to address the issues of unmelted powder particles and pores in the scaffold trabecula, which would negatively impact the scaffold's mechanical properties.

The review had certain limitations. Firstly, no standardization existed related to the pore size, porosity, strut size and pore shape among different studies. As all the parameters are correlated, thereby it was difficult to determine the best combination for designing the Ti64 scaffold. Secondly, most studies used rabbit’s femoral epiphysis as the implantation site and a 5 mm diameter scaffold, which is too large for a rabbit model and also it does not represent the functional Ti64 scaffold used in humans. However, it should be noted that a scaffold with a smaller diameter is difficult to manufacture by the available 3D printing techniques. Finally, the longest follow-up time-point reported by majority of the studies was 12 weeks; hence, it was difficult to predict whether and when the new bone would outgrow the Ti64 scaffold.

## Conclusion

Ti64 scaffold could act as a promising medium for providing mechanical support and a stable environment for new bone formation in long bone defects. Furthermore, rhombic dodecahedron- or diamond-shaped pores with a pore size of 500–700 µm and 60–70% porosity could be considered as the most optimal parameters for manufacturing the scaffold. Further studies are required using large animal models and standardized protocols for extrapolating the results of animal studies to humans for potential clinical applications.

## Supplementary Information

**Additional file 1.** The detailed search strings in this study.

**Additional file 2.** The PRISMA checklist.

## Data Availability

The datasets used and/or analyzed during the current study are available from the corresponding author on reasonable request.

## Abbreviations

Ti6Al4V: Titanium-6 aluminium-4 vanadium
Ti64 scaffold: 3D-printed porous Ti6Al4V scaffold
CAD: Computer-aided design
AM: Additive manufacturing
SLM: Selective laser melting
EBM: Electron beam melting
SLS: Selective laser sintering
PRISMA: Preferred Reporting Items for Systematic Reviews and Meta-Analyses
SEM: Scanning electron microscopy
BA/TA: Bone area fraction
BV/TV: Bone volume fraction
HA: Hydroxyapatite
BMP-2: Bone morphogenetic protein-2
VEGF: Vascular endothelial growth factor
