# Supplementary material for: 3D-printed porous Ti6Al4V scaffolds for long bone repair in animal models: a systematic review
Source: J Orthop Surg Res. 2022 Feb 2;17:68. doi: 10.1186/s13018-022-02960-6 (PMC8812248; doi:10.1186/s13018-022-02960-6)
Supplement: Supplementary file 1 — Additional file 1. The detailed search strings in this study. [file 13018_2022_2960_MOESM1_ESM.pdf]

PubMed

Concept 1: Bone/bone regeneration/bone reconstruction

"Bone and Bones"[Mesh] OR "Bone*"[tiab] OR "Bony"[tiab] OR "condyle*" [tiab] OR "Femur*"[tiab] OR "Femor*"[tiab] OR "Trochanter*" [tiab] OR "Metatars*"[tiab] OR "Tars*"[tiab] OR "Os naviculare"[tiab] OR "Calcane*"[tiab] OR "Os calcis"[tiab] OR "Os cuboideum"[tiab] OR "Talus"[tiab] OR "Tali"[tiab] OR "Astralagus"[tiab] OR "Os talare "[tiab] OR "Os trigonum*"[tiab] OR "Ossa digitorum pedi"[tiab] OR "Fibula*"[tiab] OR "Malleol*"[tiab] OR "Os peroneum"[tiab] OR "Patella*"[tiab] OR "Tibia*"[tiab] OR "Pelvic gridle*"[tiab] OR "Sacrococcyx"[tiab] OR "Acetabul*"[tiab] OR "Cotyloid Cavit*"[tiab] OR "Ilium"[tiab] OR "Ilia*"[tiab] OR "Ischi*"[tiab] OR "Pubis"[tiab] OR "Pubes"[tiab] OR "Pubic"[tiab] OR "Humer*"[tiab] OR "Tubercle*"[tiab] OR "Epitrochlea*"[tiab] OR "Anatomic neck*"[tiab] OR "Radius*"[tiab] OR "Radii*"[tiab] OR "Radial"[tiab] OR "Radical tuberosit*"[tiab] OR "Ulna*"[tiab] OR "Trochlear notch*"[tiab] OR "Semilunar notch*"[tiab] OR "Olecranon*"[tiab] OR "Clavicl*"[tiab] OR "Os Capitatum"[tiab] OR "Os Carpal"[tiab] OR "Ossa carpalia"[tiab] OR "Os hamatum"[tiab] OR "hook of the hamate*"[tiab] OR "Os lunatum"[tiab] OR "Os pisiforme"[tiab] OR "Os naviculare"[tiab] OR "Os scaphoideum"[tiab] OR "Os trapezium"[tiab] "Os multangulum "[tiab] OR "Os trapezoideum"[tiab] OR " Os trapezium minor"[tiab] OR "Triquetrum"[tiab] OR "Phalang*"[tiab] OR "Phalanx*"[tiab] OR "Metacarp*"[tiab] OR "Scapula*"[tiab] OR "Shoulder*"[tiab] OR "Acromia*"[tiab] OR "Biacromial distance*"[tiab] OR "Coracoid*"[tiab] OR "Glenoid cavit*"[tiab] OR " Glenoid fossa*"[tiab] OR "Diaphys*"[tiab] OR "Epiphys*"[tiab] OR "Metaphys"[tiab] OR "Growth plate*"[tiab] OR "Epiphyseal cartilage*"[tiab] OR "Epiphyseal plate*"[tiab] OR "Hyoid*"[tiab] OR "Thoracic cage*"[tiab] OR "Costa*"[tiab] OR "Rib"[tiab] OR "Ribs"[tiab] OR "Sternum*"[tiab] OR "Manubrium*"[tiab] OR "Manubria*"[tiab] OR "Sternal bod*"[tiab] OR "Xiphoid process*"[tiab] OR "Sesamoid*"[tiab] OR "Osteoid sesam"[tiab] OR "Skeleton*"[tiab] OR "Skeletal"[tiab] OR "Skull*"[tiab] OR "Cranium*"[tiab] OR "Crania*"[tiab] OR "Cranii"[tiab] OR "Calvari*"[tiab] OR "Bregma*"[tiab] OR "fontanel*"[tiab] OR "Suture junction*"[tiab] OR "Ossis ethmoidalis"[tiab] OR "Cribriform plate*"[tiab] OR "Jaw"[tiab] OR "Jaws"[tiab] OR "Gnathology"[tiab] OR "Alveol*"[tiab] OR "Tooth socket*"[tiab] OR "Dental socket*"[tiab] OR "Dental arch*"[tiab] OR "Tooth arch*"[tiab] OR "Arcus dentalis"[tiab] OR "Mandible*"[tiab] OR "Mandibul*"[tiab] OR "Mylohyoid ridge*"[tiab] OR "Mylohyoid groove*"[tiab] OR "Chin*"[tiab] OR "Mentum*"[tiab] OR "Mental region*"[tiab] OR "Mental foram*"[tiab] OR "Maxilla*"[tiab] OR "Palate*"[tiab] OR "incisive papilla*"[tiab] OR "Orbit*"[tiab] OR "Eye socket*"[tiab] OR "Turbinate*"[tiab] OR "Nasal concha*"[tiab] OR "Conchae nasal*"[tiab] OR "Vomer*"[tiab] OR "Zygoma*"[tiab] OR "Os frontale"[tiab] OR "Os occipitale*"[tiab] OR "Foramen magnum*"[tiab] OR "Os parietale*"[tiab] OR "Pterygo*"[tiab] OR "Basicranium*"[tiab] OR "Olfactory groove*"[tiab] OR "Posterior fossa*"[tiab] OR "Fossa posterior*"[tiab] OR "Posterior cerebral fossa*"[tiab] OR "Sphenopalatine fossa*"[tiab] OR "Clivus"[tiab] OR "Jugular foram*"[tiab] OR "Foram* jugulare*"[tiab] OR "Infratemporal*"[tiab] OR

"Parapharyng*"[tiab] OR "Para-pharyng*"[tiab] OR "Pharyng*"[tiab] OR "Spatium laterophayngeum"[tiab] OR "Styloid compartment*"[tiab] OR "Poststyloid"[tiab] OR "Prestyloid"[tiab] OR "Post-styloid"[tiab] OR "Pre-styloid"[tiab] OR "Carotid space*"[tiab] OR "Sphenoid*"[tiab] OR "Sella*"[tiab] OR "Pituitary fossa*"[tiab] OR "Hypophysis recess*"[tiab] OR "Os temporal"[tiab] OR "Facial*"[tiab] OR "Fallopi*"[tiab] OR "Stylomastoid foram*"[tiab] OR "Mastoid*"[tiab] OR "Petrous*"[tiab] OR "Spine*"[tiab] OR "Spina*"[tiab] OR "Spinous*"[tiab] OR "Column*"[tiab] OR "Backbone"[tiab] OR "Spinosus"[tiab] OR "Vertebra*"[tiab] OR "Epistropheus"[tiab] OR " Os odontoideum*"[tiab] OR "Odontoid*"[tiab] OR "Dens"[tiab] OR "Axis"[tiab] OR "Axes"[tiab] OR "atlas*"[tiab] OR "Arcuate Foramen"[tiab] OR "Ponticulus Posticus"[tiab] OR "Kimmerle anomal*"[tiab] OR "Coccyx*"[tiab] OR "Coccyg*"[tiab] OR "Tailbone*"[tiab] OR "Transverse process*"[tiab]] OR "Process transvers*"[tiab] OR "intervertebral*"[tiab] OR " Annulus fibros*"[tiab] OR "Nucleus pulposus"[tiab] OR "Nucleus fibrosus"[tiab] OR "Pulpy nucle*"[tiab] OR "Lumbar*"[tiab] OR "Lumbalis"[tiab] OR "Sacrum*"[tiab] OR "Sacra*"[tiab] OR "Epidural space*"[tiab] OR "Substantia compacta"[tiab] OR "Haversian"[tiab] OR "Osteon*"[tiab] OR "Nutrient canal*"[tiab] OR "Volkmann canal*"[tiab] OR "Perioste*"[tiab] OR "Extremities"[Mesh] OR "Extremit*"[tiab] OR "Digit*"[tiab] OR "Amputation stump*"[tiab] OR "limb*"[tiab] OR "Membrum inferius"[tiab] OR "Ankle*"[tiab] OR "Buttock*"[tiab] OR "Gluteal region*"[tiab] OR "Foot*"[tiab] OR "Feet"[tiab] OR "Forefoot"[tiab] OR "Forefeet"[tiab] OR "Forepaw*"[tiab] OR "Toe"[tiab] OR "Toes"[tiab] OR "Hallu*"[tiab] OR "Heel*"[tiab] OR "Hip"[tiab] OR "Hips"[tiab] OR "Coxa*"[tiab] OR "Knee*"[tiab] OR "Genopathy"[tiab] OR "Infrapatellar fat pad*"[tiab] OR "Crural region*"[tiab] OR "Regio cruris"[tiab] OR "Leg"[tiab] OR "Legs"[tiab] OR "Thigh*"[tiab] OR "Arm"[tiab] OR "Arms"[tiab] OR "Brachium*"[tiab] OR "Axilla*"[tiab] OR "Underarm*"[tiab] OR "Armpit*"[tiab] OR "Elbow*"[tiab] OR "Forearm*"[tiab] OR "Antebrachium*"[tiab] OR "Hand*"[tiab] OR "Finger*"[tiab] OR "Thumb*"[tiab] OR "Thenar"[tiab] OR "Wrist*"[tiab] OR "Joints"[Mesh] OR "Joint*"[tiab] OR "Articula*"[tiab] OR "Iliopsoas"[tiab] OR "Psoas"[tiab] OR "Coracoacromial"[tiab] OR "Bursa*"[tiab] OR "Tibiofibular"[tiab] OR "Planta*"[tiab] OR "Palmar*"[tiab] OR "Palma manus"[tiab] OR "Fibrocartilage*"[tiab] OR "Capsule*"[tiab] OR "Synovi*"[tiab] OR "Menisc*"[tiab] OR "Semilunar cartilage*"[tiab] OR "Ligament*"[tiab] OR "Volar plate*"[tiab] OR "Pubic symphys*"[tiab] OR "Gelnoid labrum"[tiab] OR "Temporomandibular"[tiab] OR "TMJ"[tiab] OR "Carpus"[tiab] OR "Bone substitutes"[Mesh] OR "Bone remodeling"[Mesh] OR "Osteoplasty"[tiab] OR "Osteoconduction*"[tiab] OR "Osseointegration*"[tiab] OR "Osseo-integration*"[tiab] OR "Osseous integration*"[tiab] OR "Cornerstone(device)"[tiab] OR "Hydroset"[tiab] OR "Orthopaedic endoprosthesis "[tiab] OR "Repiphysis"[tiab] OR "Endosseous healing*"[tiab] OR "Bone resorption"[Mesh] OR "Ainhum"[tiab] OR "Dactylolys*"[tiab] OR "Periodontal resorption*"[tiab] OR "Osteolys*"[tiab] OR "Acro-osteolysis"[tiab] OR "Acroosteolysis"[tiab] OR "Hajdu-cheney"[tiab] OR "Cheney syndrome*"[tiab] OR "Arthrodentoosteodysplasia*"[tiab] OR "Gorham*"[tiab] OR "Prosthetic rib system*"[tiab] OR "Extension prosthes*"[tiab] OR "Extension implant*"[tiab]

Concept 2: Titanium/Titanium alloy

"Titanium"[Mesh] OR "Titanium*"[tiab] OR "Titanum"[tiab] OR "Ti"[tiab] OR "Nitinol"[tiab]OR "Titanium alloy (TiAl6V4)" [Supplementary Concept] OR "Ti6Al4V"[tiab] OR "Ti-6Al-4V"[tiab] OR "Ti-6Al-V4"[tiab] OR "Tivanium"[tiab] OR "Tytanium"[tiab] OR "Protasul-64WF"[tiab] OR "hydroxyapatite-titanium alloy" [Supplementary Concept] OR "Hydroxy*apatite-titanium" [tiab] OR "HA-Ti" [tiab] OR "titanium-vanadium-cobalt alloy" [Supplementary Concept] OR "(Ti0.95V0.05) Co2" [tiab] OR "Ti-V-Co" [tiab] OR "titanium alloy (TiNb13Zr13)" [Supplementary Concept] OR "TiNb13Zr13" [tiab] OR "titanium-13-niobium-13-zirconium" [tiab] OR "Ti-13Nb-13Zr" [tiab] OR "Ti-6Al-7Nb alloy" [Supplementary Concept] OR "Ti-6Al-7Nb" [tiab] OR "Ti6-Al7-Nb" [tiab] OR " Ti6Al7Nb" [tiab] OR "Protasul 100" [tiab] OR "SN 56512" [tiab] OR "SN-56512" [tiab] OR "Titanium nickelide" [Supplementary Concept] OR "Ti-Ni" [tiab] OR "Nickel-titanium" [tiab] OR "Ni-Ti" [tiab] OR "Titanol" [tiab] OR "Sentalloy" [tiab] OR "Nitanium" [tiab] OR "titanium molybdenum alloy" [Supplementary Concept] OR "Ti-15Mo" [tiab] OR "titanium-niobium-aluminum alloy" [Supplementary Concept] OR "titanium-niobium-aluminum" [tiab] OR "Ti-Nb-Al" [tiab] OR "Ti-In-Nb-Ta alloy" [Supplementary Concept] OR "Ti-In-Nb-Ta" [tiab] OR "Titanium-indium-niobium-tantalum" [tiab] OR "Gold-titanium alloy" [Supplementary Concept] OR "Gold-titanium" [tiab] OR "Au-Ti" [tiab] OR "Titanium-nickel-cobalt alloy" [Supplementary Concept] OR "Titanium-nickel-cobalt" [tiab] OR "Ti(50)Ni(48.7)Co(1.3)" [tiab] OR "TiNiCo" [tiab] OR "Beta titanium" [Supplementary Concept] OR "Titanium-Niobium-Hafnium alloy" [Supplementary Concept] OR "Titanium-Niobium-Hafnium" [tiab] OR "Ti-Nb-Hf" [tiab] OR "Titanium-niobium alloy" [Supplementary Concept] OR "Titanium-niobium" [tiab] OR "Titanium aluminum alloy" [Supplementary Concept] OR "Ti5Al2.5Fe" [tiab]

Concept 3: 3D printing

"Printing, Three-Dimensional"[Mesh] OR "3D print*"[tiab] OR "3-D print*"[tiab] OR "3 D Print*"[tiab] OR "3 dimensional print*"[tiab] OR "3-dimensional print*"[tiab] OR "Three-dimensional print*"[tiab] OR "Three dimensional print*"[tiab] OR "Stereolithograph*"[tiab] OR "Stereo-lithograph*"[tiab] OR "Rapid prototyp*"[tiab] OR "Rapid proto-typ*"[tiab] OR "Additive manufactur*"[tiab] OR "Additive layer manufactur*"[tiab] OR "Fuse deposit*"[tiab] OR "Fused deposit*"[tiab] OR "FDM"[tiab] OR "Fuse filament fabricat*"[tiab] OR "Fused filament fabricat*"[tiab] OR "Fusion deposited model*"[tiab] OR "Fusion deposition model*"[tiab] OR "Powder bed fus*"[tiab] OR "Direct metal laser sinter*"[tiab] OR "DMLS"[tiab] OR "Electron beam melt*"[tiab] OR "EBM"[tiab] OR "Selective laser melt*"[tiab] OR "SLM"[tiab] OR "Selective laser sinter*"[tiab] OR "SLS"[tiab] OR "Robocast*"[tiab] OR "Robo-cast*"[tiab] OR "Direct ink writ*"[tiab] OR "DIW"[tiab] OR "SLA"[tiab] OR "Bioprint*"[tiab] OR "Bio-print*"[tiab]

Embase

Concept 1: Bone/bone regeneration/bone reconstruction

‘Bone’/exp OR ‘Bone*’:ti,ab,kw OR ‘Bony’:ti,ab,kw OR ‘Condyl*’:ti,ab,kw OR ‘Humer*’:ti,ab,kw OR ‘Epitrochlea*’:ti,ab,kw OR ‘Tubercle*’:ti,ab,kw OR ‘Anatomic neck*’:ti,ab,kw OR ‘Radius*’:ti,ab,kw OR ‘Radii’:ti,ab,kw OR ‘Radial’:ti,ab,kw OR ‘Radical tuberosit*’:ti,ab,kw OR ‘Ulna*’:ti,ab,kw OR ‘Trochlear notch*’:ti,ab,kw OR ‘Semilunar notch*’:ti,ab,kw OR ‘Olecranon*’:ti,ab,kw OR ‘Os carpale’:ti,ab,kw OR ‘Ossa carpalia’:ti,ab,kw OR ‘Os capitatum’:ti,ab,kw OR ‘Os hamatum’:ti,ab,kw OR ‘Hook of the hamate*’:ti,ab,kw OR ‘lunate*’:ti,ab,kw OR ‘Os lunatum’:ti,ab,kw OR ‘Os pisiforme’:ti,ab,kw OR ‘Os naviculare’:ti,ab,kw OR ‘Os scaphoideum’:ti,ab,kw OR ‘Os multangulum’:ti,ab,kw OR ‘Os trapezium’:ti,ab,kw OR ‘Os trapezium minor’:ti,ab,kw OR ‘Os trapezoideum’:ti,ab,kw OR ‘Triquetrum’:ti,ab,kw OR ‘Metacarp*’:ti,ab,kw OR ‘Metatars*’:ti,ab,kw OR ‘Tars*’:ti,ab,kw OR ‘Calcane*’:ti,ab,kw OR ‘Os calcis’:ti,ab,kw OR ‘Os cuboideum’:ti,ab,kw OR ‘Talus’:ti,ab,kw OR ‘Tali’:ti,ab,kw OR ‘Astralagus’:ti,ab,kw OR ‘Os talare’:ti,ab,kw OR ‘Os trigonum*’:ti,ab,kw OR ‘Ossa digitorum pedi’:ti,ab,kw OR ‘Femur*’:ti,ab,kw OR ‘Femor*’:ti,ab,kw OR ‘Trochanter*’:ti,ab,kw OR ‘Fibula*’:ti,ab,kw OR ‘Os peroneum’:ti,ab,kw OR ‘Malleol*’:ti,ab,kw OR ‘Patella*’:ti,ab,kw OR ‘Kneecap*’:ti,ab,kw OR ‘Knee cap*’:ti,ab,kw OR ‘Tibia*’:ti,ab,kw OR ‘Diaphys*’:ti,ab,kw OR ‘Epiphys*’:ti,ab,kw OR ‘Growth plate*’:ti,ab,kw OR ‘Metaphys*’:ti,ab,kw OR ‘Phalanx*’:ti,ab,kw OR ‘Phalang*’:ti,ab,kw OR ‘Sesamoid*’:ti,ab,kw OR ‘Osteoid sesam’:ti,ab,kw OR ‘Pelvic girdle’:ti,ab,kw OR ‘Sacrococcyx’:ti,ab,kw OR ‘Acetabul*’:ti,ab,kw OR ‘Cotyloid cavit*’:ti,ab,kw OR ‘Coccyx*’:ti,ab,kw OR ‘Coccyg*’:ti,ab,kw OR ‘tailbone*’:ti,ab,kw OR ‘ilium’:ti,ab,kw OR ‘ilia*’:ti,ab,kw OR ‘Ischi*’:ti,ab,kw OR ‘Pubis’:ti,ab,kw OR ‘Pubes’:ti,ab,kw OR ‘Pubic*’:ti,ab,kw OR ‘Sacrum*’:ti,ab,kw OR ‘Sacra*’:ti,ab,kw OR ‘Rib*’:ti,ab,kw OR ‘Costa*’:ti,ab,kw OR ‘Thoracic cage*’:ti,ab,kw OR ‘Shoulder*’:ti,ab,kw OR ‘Clavicl*’:ti,ab,kw OR ‘Scapula*’:ti,ab,kw OR ‘Acromion’:ti,ab,kw OR ‘Acromia*’:ti,ab,kw OR ‘Biacromial distance*’:ti,ab,kw OR ‘Coracoid*’:ti,ab,kw OR ‘Glenoid cavit*’:ti,ab,kw OR ‘Glenoid fossa*’:ti,ab,kw OR ‘Sternum*’:ti,ab,kw OR ‘Manubrium*’:ti,ab,kw OR ‘Manubria*’:ti,ab,kw OR ‘Stern* bod*’:ti,ab,kw OR ‘Xiphoid process*’:ti,ab,kw OR ‘Skeleton*’:ti,ab,kw OR ‘Skeletal’:ti,ab,kw OR ‘Skull*’:ti,ab,kw OR ‘Skull*’:ti,ab,kw OR ‘Crania*’:ti,ab,kw OR ‘Cranium*’:ti,ab,kw OR ‘Cranii’:ti,ab,kw OR ‘Alveol*’:ti,ab,kw OR ‘Olfactory groove*’:ti,ab,kw OR ‘Bregma*’:ti,ab,kw OR ‘Suture junction*’:ti,ab,kw OR ‘Calvari*’:ti,ab,kw OR ‘Clivus’:ti,ab,kw OR ‘Ossis ethmoidalis’:ti,ab,kw OR ‘Cribriform plate*’:ti,ab,kw OR ‘Fontanel*’:ti,ab,kw OR ‘Foramen magnum*’:ti,ab,kw OR ‘Os frontale’:ti,ab,kw OR ‘Hyoid*’:ti,ab,kw OR ‘Infratemporal*’:ti,ab,kw OR ‘Jaw*’:ti,ab,kw OR ‘Gnathology’:ti,ab,kw OR ‘Tooth socket*’:ti,ab,kw OR ‘Dental socket*’:ti,ab,kw OR ‘Mandible*’:ti,ab,kw OR ‘Mandibul*’:ti,ab,kw OR ‘Mylohyoid ridge*’:ti,ab,kw OR ‘Mylohyoid groove*’:ti,ab,kw OR ‘Chin*’:ti,ab,kw OR ‘Mentum*’:ti,ab,kw OR ‘Mental region*’:ti,ab,kw OR ‘Mental foram*’:ti,ab,kw OR ‘Mastoid*’:ti,ab,kw OR ‘Maxilla*’:ti,ab,kw OR ‘Palate*’:ti,ab,kw OR ‘Incisive papilla*’:ti,ab,kw OR ‘Nasal concha*’:ti,ab,kw OR ‘Conchae nasal*’:ti,ab,kw OR ‘Os

occipitale’:ti,ab,kw OR ‘Orbit*’:ti,ab,kw OR ‘Eye socket*’:ti,ab,kw OR ‘Os parietale’:ti,ab,kw OR ‘Pertros*’:ti,ab,kw OR ‘Posterior fossa*’:ti,ab,kw OR ‘Fossa posterior’:ti,ab,kw OR ‘Posterior cerebral fossa*’:ti,ab,kw OR ‘Pterygopalatin*’:ti,ab,kw OR ‘Sphenopalatine fossa*’:ti,ab,kw OR ‘Sella*’:ti,ab,kw OR ‘Pituitary fossa*’:ti,ab,kw OR ‘Hypophysis recess*’:ti,ab,kw OR ‘Pharyng*’:ti,ab,kw OR ‘Parapharyng*’:ti,ab,kw OR ‘Para-pharyng*’:ti,ab,kw OR ‘Pterygo*’:ti,ab,kw OR ‘Spatium lateropharyngeum’:ti,ab,kw OR ‘Basicranium*’:ti,ab,kw OR ‘Styloid compartment*’:ti,ab,kw OR ‘Prestyloid’:ti,ab,kw OR ‘Pre-styloid’:ti,ab,kw OR ‘Poststyloid’:ti,ab,kw OR ‘Post-styloid’:ti,ab,kw OR ‘Carotid space*’:ti,ab,kw OR ‘Sphenoid*’:ti,ab,kw OR ‘Os temporal’:ti,ab,kw OR ‘Facial*’:ti,ab,kw OR ‘Fallopi*’:ti,ab,kw OR ‘Stylomastoid foram*’:ti,ab,kw OR ‘Jugular foram*’:ti,ab,kw OR ‘Foram* jugulare*’:ti,ab,kw OR ‘Tooth arch*’:ti,ab,kw OR ‘Dental arch*’:ti,ab,kw OR ‘Arcus dentalis’:ti,ab,kw OR ‘Turbinate*’:ti,ab,kw OR ‘Vomer*’:ti,ab,kw OR ‘Zygoma*’:ti,ab,kw OR ‘Spine*’:ti,ab,kw OR ‘Spina*’:ti,ab,kw OR ‘Spinous’:ti,ab,kw OR ‘Column*’:ti,ab,kw OR ‘Vertebra*’:ti,ab,kw OR ‘backbone*’:ti,ab,kw OR ‘Epidural space*’:ti,ab,kw OR ‘atlas’:ti,ab,kw OR ‘Arcuate foramen’:ti,ab,kw OR ‘Ponticulus posticus’:ti,ab,kw OR ‘Kimmerle anomal*’:ti,ab,kw OR ‘axis’:ti,ab,kw OR ‘axes’:ti,ab,kw OR ‘Epistropheus’:ti,ab,kw OR ‘Processus spinosus’:ti,ab,kw OR ‘Process* transvers*’:ti,ab,kw OR ‘Transvers* process*’:ti,ab,kw OR ‘Intervertebral*’:ti,ab,kw OR ‘Annulus fibros*’:ti,ab,kw OR ‘Nucleus fibrosus*’:ti,ab,kw OR ‘Nucleus pulposus*’:ti,ab,kw OR ‘Pulpy nucle*’:ti,ab,kw OR ‘Lumbar*’:ti,ab,kw OR ‘lumbalis’:ti,ab,kw OR ‘Odontoid*’:ti,ab,kw OR ‘Dens’:ti,ab,kw OR ‘Limb’/exp OR ‘Limb*’:ti,ab,kw OR ‘Extremit*’:ti,ab,kw OR ‘Digit*’:ti,ab,kw OR ‘Amputation stump*’:ti,ab,kw OR ‘Membrum inferius’:ti,ab,kw OR ‘Finger*’:ti,ab,kw OR ‘Thumb*’:ti,ab,kw OR ‘Toe’:ti,ab,kw OR ‘Toes’:ti,ab,kw OR ‘Hallu*’:ti,ab,kw OR ‘Hip’:ti,ab,kw OR ‘Hips’:ti,ab,kw OR ‘Coxa*’:ti,ab,kw OR ‘Leg’:ti,ab,kw OR ‘Legs’:ti,ab,kw OR ‘Ankle*’:ti,ab,kw OR ‘Buttock*’:ti,ab,kw OR ‘Gluteal region*’:ti,ab,kw OR ‘Foot’:ti,ab,kw OR ‘Feet’:ti,ab,kw OR ‘Footpad*’:ti,ab,kw OR ‘Planta*’:ti,ab,kw OR ‘Forefoot’:ti,ab,kw OR ‘Forefeet’:ti,ab,kw OR ‘Forepaw*’:ti,ab,kw OR ‘Heel*’:ti,ab,kw OR ‘Knee*’:ti,ab,kw OR ‘Genopathy’:ti,ab,kw OR ‘Infrapatellar fat pad*’:ti,ab,kw OR ‘Crural region*’:ti,ab,kw OR ‘Regio cruris’:ti,ab,kw OR ‘Thigh*’:ti,ab,kw OR ‘Arm’:ti,ab,kw OR ‘Arms’:ti,ab,kw OR ‘Brachium*’:ti,ab,kw OR ‘Axilla*’:ti,ab,kw OR ‘Underarm*’:ti,ab,kw OR ‘Armpit*’:ti,ab,kw OR ‘Elbow’:ti,ab,kw OR ‘Forearm*’:ti,ab,kw OR ‘Antebrachium*’:ti,ab,kw OR ‘Hand*’:ti,ab,kw OR ‘Palma manus’:ti,ab,kw OR ‘Palmar*’:ti,ab,kw OR ‘Thenar’:ti,ab,kw OR ‘Wrist*’:ti,ab,kw OR ‘Joint’/exp OR ‘Joint*’:ti,ab,kw OR ‘Articula*’:ti,ab,kw OR ‘Iliopsoas’:ti,ab,kw OR ‘Psoas’:ti,ab,kw OR ‘Coracoacromial’:ti,ab,kw OR ‘Tibiofibular’:ti,ab,kw OR ‘Fibrocartilage*’:ti,ab,kw OR ‘Ligament*’:ti,ab,kw OR ‘Capsule’:ti,ab,kw OR ‘Volar plate*’:ti,ab,kw OR ‘Menisc*’:ti,ab,kw OR ‘Bursa*’:ti,ab,kw OR ‘Synovi*’:ti,ab,kw OR ‘Semilunar cartilage’:ti,ab,kw OR ‘Pubic symphys’:ti,ab,kw OR ‘Gelnoid labrum’:ti,ab,kw OR ‘Temporomandibular’:ti,ab,kw OR ‘TMJ’:ti,ab,kw OR ‘Carpus’:ti,ab,kw OR ‘Bone remodeling’/exp OR ‘Osteoplasty’:ti,ab,kw OR ‘Bone regeneration’/exp OR ‘Osseointegration*’:ti,ab,kw OR ‘Osseo-integration*’:ti,ab,kw OR ‘Osseous integration*’:ti,ab,kw OR ‘Osteoconduction*’:ti,ab,kw OR ‘Endosseous healing’:ti,ab,kw OR

‘Ainhum’:ti,ab,kw OR ‘Dactylolys*’:ti,ab,kw OR ‘Periodontal resorption*’:ti,ab,kw OR ‘Osteolys*’:ti,ab,kw OR ‘Acro-osteolys*’:ti,ab,kw OR ‘Acroosteolys*’:ti,ab,kw OR 'Bone tissue engineering'/exp OR 'Bone prosthesis'/exp OR ‘Cornerstone(device)’:ti,ab,kw OR ‘Hydroset’:ti,ab,kw OR ‘Orthop* endoprosthesis’:ti,ab,kw OR ‘Repiphysis’:ti,ab,kw OR ‘Extension implant*’:ti,ab,kw OR ‘Extension prosthes*’:ti,ab,kw OR ‘Substantia compacta*’:ti,ab,kw OR ‘Haversian’:ti,ab,kw OR ‘Osteon*’:ti,ab,kw OR ‘Nutrient canal*’:ti,ab,kw OR ‘Volkmann canal*’:ti,ab,kw OR ‘Perioste*’:ti,ab,kw OR ‘Hajdu-cheney’:ti,ab,kw OR ‘Cheney syndrome’:ti,ab,kw OR ‘Arthrodentoosteodysplasia*’:ti,ab,kw OR ‘Gorham*’:ti,ab,kw

Concept 2: titanium/titanium alloy
‘Titanium’/exp OR ‘Titanium*’:ti,ab,kw OR ‘Titanum’:ti,ab,kw OR ‘Ti’:ti,ab,kw OR ‘Titanium alloy tial6v4’/exp OR ‘Titanium alloy tinb13zr13’/exp OR ‘Nitinol’/exp OR ‘Nitinol’:ti,ab,kw OR ‘Titanium derivative’/exp OR ‘Ti6Al4V’:ti,ab,kw OR ‘Ti-6Al-4V’:ti,ab,kw OR ‘Ti-6Al-V4’:ti,ab,kw OR ‘Tivanium’:ti,ab,kw OR ‘Tytanium’:ti,ab,kw OR ‘Protasul-64WF’:ti,ab,kw OR ‘Hydroxy*apatite-titanium’:ti,ab,kw OR ‘HA-Ti’:ti,ab,kw OR ‘(Ti0.95V0.05) Co2’:ti,ab,kw OR ‘Ti-V-Co’:ti,ab,kw OR ‘TiNb13Zr13’:ti,ab,kw OR ‘Ti-13Nb-13Zr’:ti,ab,kw OR ‘Ti-6Al-7Nb’:ti,ab,kw OR ‘Ti6-Al7-Nb’:ti,ab,kw OR ‘Ti6Al7Nb’:ti,ab,kw OR ‘Protasul 100’:ti,ab,kw OR ‘SN 56512’:ti,ab,kw OR ‘SN-56512’:ti,ab,kw OR ‘Ti-Ni’:ti,ab,kw OR ‘Nickel-titanium’:ti,ab,kw OR ‘Ni-Ti’:ti,ab,kw OR ‘Titanol’:ti,ab,kw OR ‘Sentalloy’:ti,ab,kw OR ‘Nitanium’:ti,ab,kw OR ‘Ti-15Mo’:ti,ab,kw OR ‘Ti-Nb-Al’:ti,ab,kw OR ‘Ti-In-Nb-Ta’:ti,ab,kw OR ‘Gold-titanium’:ti,ab,kw OR ‘Au-Ti’:ti,ab,kw OR ‘Ti(50)Ni(48.7)Co(1.3)’:ti,ab,kw OR ‘TiNiCo’:ti,ab,kw OR ‘Ti-Nb-Hf’:ti,ab,kw OR ‘Ti5Al2.5Fe’:ti,ab,kw

Concept 3: 3d printing
‘Rapid prototyping’/exp OR ‘Rapid prototyp*’:ti,ab,kw OR ‘Rapid proto-typ*’:ti,ab,kw OR ‘Three dimensional print*’:ti,ab,kw OR ‘Three-dimensional print*’:ti,ab,kw OR ‘3 dimensional print*’:ti,ab,kw OR ‘3-dimensional print*’:ti,ab,kw OR ‘3-D print*’:ti,ab,kw OR ‘3D print*’:ti,ab,kw OR ‘3 D print*’:ti,ab,kw OR ‘Additive manufactur*’:ti,ab,kw OR ‘Additive layer manufactur*’:ti,ab,kw OR ‘Fuse* deposit* model*’:ti,ab,kw OR ‘FDM’:ti,ab,kw OR ‘Fuse* filament fabricat*’:ti,ab,kw OR ‘Fusion deposit* model*’:ti,ab,kw OR ‘Powder bed fus*’:ti,ab,kw OR ‘Direct metal laser sinter*’:ti,ab,kw OR ‘DMLS’:ti,ab,kw OR ‘Electron beam melt*’:ti,ab,kw OR ‘EBM’:ti,ab,kw OR ‘Selective laser melt*’:ti,ab,kw OR ‘SLM’:ti,ab,kw OR ‘Selective laser sinter*’:ti,ab,kw OR ‘SLS’:ti,ab,kw OR ‘Robocast*’:ti,ab,kw OR ‘Robo-cast*’:ti,ab,kw OR ‘Direct ink writ*’:ti,ab,kw OR ‘DIW’:ti,ab,kw OR ‘Stereolithograph*’:ti,ab,kw OR ‘SLA’:ti,ab,kw OR ‘Stereo-lithography’:ti,ab,kw OR ‘Bioprinting’:ti,ab,kw OR ‘Bio-printing’:ti,ab,kw

Web of science

Concept 1: Bone/bone regeneration/bone reconstruction

"Bone*" OR "Bony" OR "condyle*" OR "Bone-implant interface*" OR "Bone-prosthesis interface*" OR "Femur*" OR "Femor*" OR "Trochanter*" OR "Ligamentum teres of the hip*" OR "Round ligament of the hip*" OR "Metatars*" OR "Tars*" OR "Os naviculare" OR "Calcane*" OR "Os calcis" OR "Os cuboideum" OR "Talus" OR "Tali" OR "Astralagus" OR "Os talare " OR "Os trigonum*" OR "Ossa digitorum pedi" OR "Fibula*" OR "Malleol*" OR "Os peroneum" OR "Patella*" OR "Kneecap*" OR "Knee cap" OR "Tibia*" OR "Pelvic gridle*" OR "Pelvic limb*" OR "Sacrococcyx" OR "Acetabul*" OR "Cotyloid Cavit*" OR "Ilium" OR "Ilia*" OR "Ischi*" OR "Pubis" OR "Pubes" OR "Pubic" OR "Humer*" OR "Tubercle*" OR "Epitrochlea*" OR "Anatomic neck*" OR "Radius*" OR "Radii*" OR "Radial" OR "Radical tuberosit*" OR "Ulna*" OR "Trochlear notch*" OR "Semilunar notch*" OR "Olecranon*" OR "Clavicl*" OR "Os Capitatum" OR "Os Carpal" OR "Ossa carpalia" OR "Os hamatum" OR "hook of the hamate*" OR "Os lunatum" OR "Os pisiforme" OR "Os naviculare" OR "Os scaphoideum" OR "Os trapezium" OR "Os multangulum" OR "Os trapezoideum" OR "Os trapezium minor" OR "Triquetrum" OR "Phalang*" OR "Phalanx*" OR "Metacarp*" OR "Scapula*" OR "Shoulder*" OR "Acromia*" OR "Biacromial distance*" OR "Coracoid*" OR "Glenoid cavit*" OR " Glenoid fossa*" OR "Diaphys*" OR "Epiphys*" OR "Metaphys" OR "Growth plate*" OR "Epiphyseal cartilage*" OR "Epiphyseal plate*" OR "Hyoid*" OR "Thoracic cage*" OR "Costa*" OR "Rib" OR "Ribs" OR "Sternum*" OR "Manubrium*" OR "Manubria*" OR "Stern* bod*" OR "Xiphoid process*" OR "Sesamoid*" OR "Osteoid sesam" OR "Skeleton*" OR "Skeletal" OR "Skull*" OR "Cranium*" OR "Crania*" OR "Cranii" OR "Calvari*" OR "Bregma*" OR "fontanel*" OR "Suture junction*" OR "Ossis ethmoidalis" OR "Cribriform plate*" OR "Jaw" OR "Jaws" OR "Gnathology" OR "Alveol*" OR "Tooth socket*" OR "Dental socket*" OR "Dental arch*" OR "Tooth arch*" OR "Arcus dentalis" OR "Mandible*" OR "Mandibul*" OR "Mylohyoid ridge*" OR "Mylohyoid groove*" OR "Chin*" OR "Mentum*" OR "Mental region*" OR "Mental foram*" OR "Maxilla*" OR "Palate*" OR "incisive papilla*" OR "Orbit*" OR "Eye socket*" OR "Turbinate*" OR "Nasal concha*" OR "Conchae nasal*" OR "Vomer*" OR "Zygoma*" OR "Os frontale" OR "Os occipitale*" OR "Foramen magnum*" OR "Os parietale*" OR "Pterygo*" OR "Basicranium*" OR "Olfactory groove*" OR "Posterior fossa*" OR "Fossa posterior*" OR "Posterior cerebral fossa*" OR "Sphenopalatine fossa*" OR "Clivus" OR "Jugular foram*" OR "Foram* jugulare*" OR "Infratemporal*" OR "Parapharyng*" OR "Para-pharyng*" OR "Pharyng*" OR "Spatium lateropharyngeum" OR "Styloid compartment*" OR "Poststyloid" OR "Prestyloid" OR "Post-styloid" OR "Pre-styloid" OR "Carotid space*" OR "Sphenoid*" OR "Sella*" OR "Pituitary fossa*" OR "Hypophysis recess*" OR "Os temporal" OR "Facial*" OR "Fallopi*" OR "Stylomastoid foram*" OR "Mastoid*" OR "Petrous*" OR "Spine*" OR "Spina*" OR "Spinous*" OR "Column*" OR "Backbone" OR "Spinosus" OR "Vertebra*" OR "Epistropheus" OR " Os odontoideum*" OR "Odontoid*" OR "Dens" OR "Axis" OR "Axes" OR "atlas*" OR "Arcuate Foramen" OR "Ponticulus Posticus" OR "Kimmerle anomal*" OR "Coccyx*" OR

"Coccyg*" OR "Tailbone*" OR "Transvers* process*" OR "Process* transvers*" OR "intervertebral*" OR " Annulus fibros*" OR "Nucleus pulposus" OR "Nucleus fibrosus" OR "Pulpy nucle*" OR "Lumbar*" OR "Lumbalis" OR "Sacrum*" OR "Sacra*" OR "Epidural space*" OR "Substantia compacta" OR "Haversian" OR "Osteon*" OR "Nutrient canal*" OR "Volkmann canal*" OR "Perioste*" OR "Extremit*" OR "Digit*" OR "Amputation stump*" OR "limb*" OR "Membrum inferius" OR "Ankle*" OR "Buttock*" OR "Gluteal region*" OR "Foot*" OR "Feet" OR "Forefoot" OR "Forefeet" OR "Forepaw*" OR "Toe" OR "Toes" OR "Hallu*" OR "Heel*" OR "Hip" OR "Hips" OR "Coxa*" OR "Knee*" OR "Genopathy" OR "Infrapatellar fat pad*" OR "Crural region*" OR "Regio cruris" OR "Leg" OR "Legs" OR "Thigh*" OR "Arm" OR "Arms" OR "Brachium*" OR "Axilla*" OR "Underarm*" OR "Armpit*" OR "Elbow*" OR "Forearm*" OR "Antebrachium*" OR "Hand*" OR "Finger*" OR "Thumb*" OR "Thenar" OR "Wrist*" OR "Joint*" OR "Articula*" OR "Iliopsoas" OR "Psoas" OR "Coracoacromial" OR "Bursa*" OR "Tibiofibular" OR "Planta*" OR "Palmar*" OR "Palma manus" OR "Fibrocartilage*" OR "Capsule*" OR "Synovi*" OR "Menisc*" OR "Semilunar cartilage*" OR "Ligament*" OR "Volar plate*" OR "Pubic symphys*" OR "Gelnoid labrum" OR "Temporomandibular" OR "TMJ" OR "Carpus" OR "Osteoplasty" OR "Osteoconduction*" OR "Osseointegration*" OR "Osseo-integration*" OR "Osseous integration*" OR "Cornerstone(device)" OR "Hydroset" OR "Orthop* endoprosthesis " OR "Repiphysis" OR "Endosseous healing*" OR "Ainhum" OR "Dactylolys*" OR "Periodontal resorption*" OR "Osteolys*" OR "Acro-osteolysis" OR "Acroosteolysis" OR "Hajdu-cheney" OR "Cheney syndrome*" OR "Arthrodentoosteodysplasia*" OR "Gorham*" OR "Prosthetic rib system*" OR "Extension prosthes*" OR "Extension implant*"

Concept 2: Titanium/Titanium alloy

"Titanium*" OR "Titanum" OR "Ti" OR "Nitinol" OR "Ti6Al4V" OR "Ti-6Al-4V" OR "Ti-6Al-V4" OR "Tivanium" OR "Tytanium" OR "Protasul-64WF" OR "Hydroxy*apatite-titanium" OR "HA-Ti" OR "(Ti0.95V0.05) Co2" OR "Ti-V-Co" OR "TiNb13Zr13" OR "titanium-13-niobium-13-zirconium" OR "Ti-13Nb-13Zr" OR "Ti-6Al-7Nb" OR "Ti6-Al7-Nb" OR " Ti6Al7Nb" OR "Protasul 100" OR "SN 56512" OR "SN-56512" OR "Ti-Ni" OR "Nickel-titanium" OR "Ni-Ti" OR "Titanol" OR "Sentalloy" OR "Nitanium" OR "Ti-15Mo" OR "titanium-niobium-aluminum" OR "Ti-Nb-Al" OR "Ti-In-Nb-Ta" OR "Titanium-indium-niobium-tantalum" OR "Gold-titanium" OR "Au-Ti" OR "Titanium-nickel-cobalt" OR "Ti(50)Ni(48.7)Co(1.3)" OR "TiNiCo" OR "Titanium-Niobium-Hafnium" OR "Ti-Nb-Hf" OR "Titanium-niobium" OR "Ti5Al2.5Fe"

Concept 3: 3D printing

"3D print*" OR "3-D print*" OR "3 D Print*" OR "3 dimensional print*" OR "3-dimensional print*" OR "Three-dimensional print*" OR "Three dimensional print*" OR "Stereolithograph*" OR "Stereo-lithograph*" OR "Rapid prototyp*" OR "Rapid proto-typ*"

OR "Additive manufactur*" OR "Additive layer manufactur*" OR "Fuse deposit*" OR "Fused deposit*" OR "FDM" OR "Fuse filament fabricat*" OR "Fused filament fabricat*" OR "Fusion deposited model*" OR "Fusion deposition model*" OR "Powder bed fus*" OR "Direct metal laser sinter*" OR "DMLS" OR "Electron beam melt*" OR "EBM" OR "Selective laser melt*" OR "SLM" OR "Selective laser sinter*" OR "SLS" OR "Robocast*" OR "Robo-cast*" OR "Direct ink writ*" OR "DIW" OR "SLA" OR "Bioprint*" OR "Bio-print*"
